# A computational study of a chemical gas sensor utilizing Pd–rGO composite on SnO_2_ thin film for the detection of NO_x_

**DOI:** 10.1038/s41598-020-78586-7

**Published:** 2021-01-13

**Authors:** S. Akshya, A. Vimala Juliet

**Affiliations:** Department of Electronics and Instrumentation Engineering, SRM Institute of Science and Technology, Kattankulathur 603203, Tamil Nadu India

**Keywords:** Environmental sciences, Chemistry, Engineering, Materials science, Mathematics and computing, Optics and photonics, Physics

## Abstract

In this paper we discussed, nitrogen oxides gas sensors are designed and simulated using the MEMS-based tool of COMSOL Multiphysics software. Pd–rGO composite films were designed and their NO_x_ sensing characteristics were investigated in this study by comparing with/without active layers. Transition metal SnO_2_ deals with four different active materials i.e., Pure SnO_2_, SnO_2_–Pd, SnO_2_–rGO, and SnO_2_–Pd/rGO film was controlled by altering the active materials during the active layer deposition. The deposition of Pd/rGO active material is integrated into the SnO_2_ thin film. The response of the nanocomposite materials on the NO_x_ gas sensor at a low temperature below 100 °C was significantly improved. Moreover, we investigate the optimization from different active layer response for NO_x_ by applying power in watt and milliwatt to the interdigitated electrode on the Sn substrate. The determination is tense to finalize the suitable materials that to detect more response for nitrogen oxides i.e., Pd/rGO layer shows better performance when compared with other active layers for the sensing of nitrogen oxides is in proportion to the power in the range of 0.6–4.8 W at (1–8) Voltage range. This advanced research will enable a new class of portable NO_x_ gas sensors to be constructed with millimeter size and microwatt power.

## Introduction

Chemical gas sensors hinge on oxide matters are among the majority of shapes for the modelling of sensors. Metal oxide gas sensor were developed in synthesis technique of doping two dimensional active material . To improve sensitivity of metal oxide sensor at low operating temperature were focused and performed. Studies involved on surface photoactive of sensing layer considered as the compatible material in the architecture of the device. They fabricated sensor using the 2 mm × 2 mm × 0.254 mm alumina substrate and platinum as a lead. The electrical characteristics of transition metal oxides are changed due to the adsorption/desorption processes of gases analytes on their surface of the electrode^1^. Chemical gas sensors are hugely used in fields such as environmental pollutant monitoring, industrial safety measures, and universal security. Even a small percentage of pollutant gases like NO_x_, CO, SO_x_, etc. Here gas sensor is defined by communication between chemical species along with surface of the active layers. When the active surface layer exposed to target chemical species will reacts and changes the characteristics of the chemical gas sensor. The aim of this project involves in identifying the different thickness of the heater design for sensing VOC’s to the uniform heat supplied on the whole sensor by conduction. Mender shape micro heater was analysed and also its provides uniform temperature distribution. Platinum is chosen material for sensing and gives linear results for thickness (1.5 μm to 5 μm) of versus temperature (45 °C to 49 °C)^2^. May be vicious to human health. Chemical gas sensors are the optimistic devices that gives the effective sensing and huge quality. Metal oxide gas sensor are promising device which gives good sensitivity and selectivity over target gas molecules. A real time testing setup is required to identify its reliability and worthiness. Therefore, its necessary to optimize the sensing chamber which can fit in to testing and develop the sensing and acting on target gases of the gas sensor. Simulated results we can observe that designed and optimized for substrate position and inlet/outlet walls of the gas chamber^3^. Also, chemical-based sensors are mostly suitable for an environmental pollutant gas sensor. Microsensors have come out as a universal, powerful, and great things to investigate scientific occurrence. MEMS has evolved exponentially previous two decades and in this survey it was focused on finite element modelling analysis of three layer which is nearly related to mathematical model. The main theme of this work was to analysis of effects of angle of inclination of the applied load, relationship between load vs deformation due to stress, strain and level of accuracy. Three different materials were used to find out the better stress, they are silicon dioxide, polysilicon and nitride. The results from cantilever deflection was considered for the biomedical application^4^. In this segment, we briefly presented the working concept and the significance of chemical gas sensors. Palladium is a well known alloy or noble metal, playing much important role in industrial applications due to its noble synthesized and solid properties. Its improving attention due to the high corrosion resistance, high catalytic activity, withstand and high gas absorption capacity. Palladium have been emerged as a great replacement material to metal oxide. Since, palladium can exhibit both gas response and high sensing/selectivity towards few gases such as nitrogen dioxide, carbon monoxide, hydrogen etc., But it gives more response towards nitrogen dioxide with its active particles such as rGO, tin, zinc etc. The synthesis of nanostructured palladium (nanoparticles) can improve its catalytic activity. Graphene has confirmed merits over the gas sensing materials. Its is ideally two dimensional atomic material and has extreme aspect area to its capacity ratio. Also. Now graphene have grouped great interest as an suporting material because of their high surface area 2630 m^2^/g, high electron mobility 200,000 cm^2^ v^−1^ s^−1^, high chemical stability and high conductivity 10^3^–10^4^ s/m. Its physical and chemical characteristics gives huge advantages in the electrochemical sensors. Therefore, the reduced graphene oxide is reduced from graphene oxide also when its deposited on the conductive electrode will increase its stability due to its parameter such as concentration, current density and scan rate. Hence, the rGO is deposited on palladium deposited layer is a promising combination for nitrogen dioxide sensing material due to its large surface area for gas molecules adsorption and excellent electrical properties of Pd–rGO such as high carrier mobility. Simulated results with Pd–rGO sensing materials, such as rGO is deposited on Pd deposited layer on the tin substrate were solved for NO_x_ sensors. This analytical studies also revealed the mechanism of gas molecules adsorption onto Pd–rGO actives layers.

**Figure 1:**
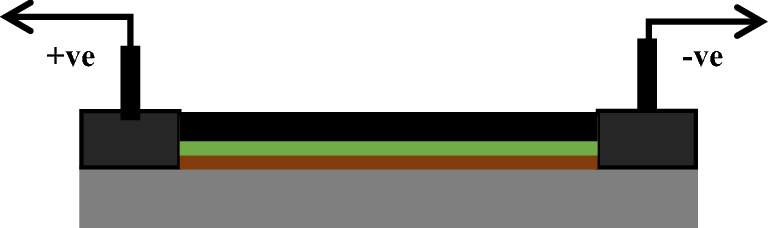
Schematic of a cross sectional view of a Pd/rGO gas sensor.

**Figure 2:**
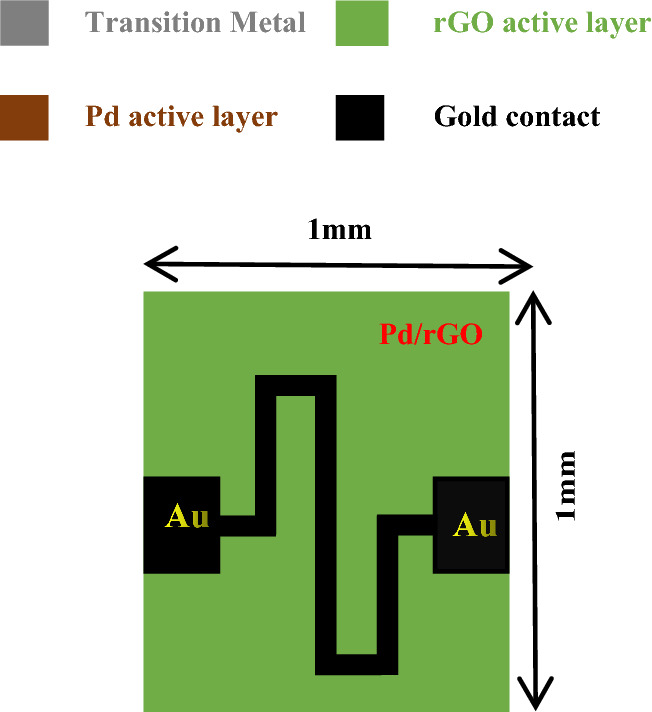
Top view of chemical gas sensor with Pd/rGO active layers.

Deposition of nanocomposites on the transition metal to act as an active materials. It's important to consider the choice of the substrate material that should withstand temperature and environmental conditions. In a particular way, we zoomed on the tasks for the deposition of active materials Pd/rGO layer by layer on the electrode. We discussed the enhancement of the sensing act of the modeled sensor and to optimize the active materials at different operating temperatures. Among environmental pollutant gases, NO_x_ is one of the major harmful gases emerging from various sources such as domestic, chemical industry, automation, and critical environmental. Current sensors are not convenient for continual air quality monitoring due to high power, slow response, and low sensitivity. In this paper, they have chosen AlGaN/GaN high electron mobility transistor i.e., HEMT sensor self-heating are modeled and studied. When the modeled sensor is exposed to concentration of 100 ppb NO_2_/NO at 300 °C. Also this sensor exposed to 1 ppb, which gives high sensitivity of 1.1% is obtained. This type of sensor is suitable for continuous monitoring of environmental pollutant at high temperature^5^. In this paper, a design of Tin dioxide with/without a Pd/rGO active layer for the NO_x_ gas sensor with IDE is presented. Aluminium and gallium materials usually preferred as heterojunctions that gives the high potential for all sensors. Due to its high density state (2DEG) at the interface, which gives a huge response. They have calculated the polarized induced charges bind between AlGaN/GaN interface from various sets of piezoelectric constants between the AlN and GaN chemical compound. Especially when the identified strain on piezoelectric that induces the charges on the sheet could be calculated with accuracy of 20%. The Non-linear stress found to be neglected^6^. This paper solved finite element method is used to get the better performance of a piezoresistive microcantilever based sensor and optimized the best design based on various load. The sensitivity was performed by changing both cantilever and piezoresistive thickness from minimum to maximum range. Simulated result shows that the sensor sensitivity is maximum at minimum thickness of both piezoresistive and cantilever. Then comparison was performed between Polysilicon and SiO_2_ material. The SCR cantilever of 0.2 µm thickness gives extreme tension which results in high response towards target^7^ high deflect in for the same applied load. Designed 2D chemical gas sensors, as shown from Figs. 1 and 2, have important parameters such as selectivity, sensitivity, and response time of gas sensors that can be improved by increasing the surface temperature.

MEMS based Sensor gets disturbed to substantial impact from environs aspects are humidity, temperature and other air pollutants in atmosphere. Acknowledging these factors, we simulated experiments to analysis target gas molecules in the form of load and simulated results were considered as a response for target gas pollutant. Also, we have to carry out all this factor in the real world applications through a practical experiment of the gas sensor calibration worn to turn the sensor response to the particular gas concentration and to right the factors on sensor output and increase the device stability and quality over such factors. Here, for the first time, we have designed suspended Pd/rGO NO_x_ sensors on SnO_2_ thin-film with IDE as a sensor platform. The sensor comprises a Pd/rGO layers suspended within a tin dioxide film. A SnO_2_ thin film provides an effective material for NO_x_ gas sensing incorporating with actives layers and the tin dioxide film for reduction of power consumption, low to 0.6–4.8 W when the active layers of the sensor are interacting with nitrogen and oxygen gas molecules at particular temperature. Conducting and aspect such as electrical potential, Charge density, volumetric stress of the Pd/rGO on the surface of the tin substrate are studied for the first time by using COMSOL Multiphysics. Temperature and sensible enthalpy response on Pd/rGO material gas sensor device are explored and finalized to nitrogen oxides gas are presented. We conclude that the Pd/rGO–Tin sensor concept that combines the gas sensor with an IDE can be applied to Nitrogen oxides gas sensing in many electrical, electronic and industrial applications.

This work mainly approached on the modeling and optimization of with/without active layer which should oblige evaluate of the developed chemical gas sensor for sensitivity and selectivity. The design is required to be finalize in the condition of with/without active layers. Figure 1 shows the cross-sectional view of a Pd/rGO chemical gas sensor. Cross sectional view and each layer was distinguished with different colors and mentioned clearly in Fig. 1. The next top view of the designed gas sensor is shown clearly with (Au) leads Fig. 2.

## Evolution of sensing structures

The substrate selects in this work for the nitrogen oxides sensing has analytic testing on the production of new compact and ambulant chemical gas sensors. Dimensions and parameters such as low cost, good bio-acceptability, oxidation resist ability, thermal stability and chemical durability must be considered to choose exact active material. They enlarge the demand for low power, dense, gas sensors for chemical industry and domestic applications direct the research of new technologies to the smallest and miniaturization of the sensor without immolating sensitivity. Amid environmental polluting gases, nitrogen oxides (NO_x_) are the most dangerous gases originating mainly from the combustion of automobile exhaust from 0.1 up to 50 ppm. In this paper, they have done the performance of Nitrogen dioxide device based on nano composite zinc oxide at working temperature of 200 °C. Here the device were developed by spin coating process and synthesized sample were analyzed by SEM and XRD that reveals the morphological analysis like nanopartcile size, shape and orientation. Also the fabricated NO_2_ gas sensor shows the sensitivity towards cross like H_2_S, Cl_2_ and NH_3._ Firstly, NO_2_ sensor film was annealed at 700 °C to gives the maximum sensing upto 78%. Sensing characteristics were carried out by using low concentration of NO_2_ from 10–100 ppm and the sensitivity was very high for NO_2_ when compared to other cross gases molecules. Hence ZnO thin film shows maximum response of 37.2% to 100 ppm of nitrogen dioxide at operating temperature of 200 °C^8^. Pt/GaN schottky diode was fabricated and were performed sensitivity test towards NO in the background of nitrogen. When the fabricated sensor exposed to nitrogen in presence of constant forward current of 10 mA in diode gives increase in voltage exponentially and decrease while nitrogen replaced by hydrogen. The response was recorded as many pulses and attain saturation gradually once hydrogen supply in to diode. The thickness of Pt/GaN Schottky diode is 40 nm that yields 0.4 mV of response voltage at concentration of 500 ppm^9^.

### Fundamental device design

**Figure 3:**
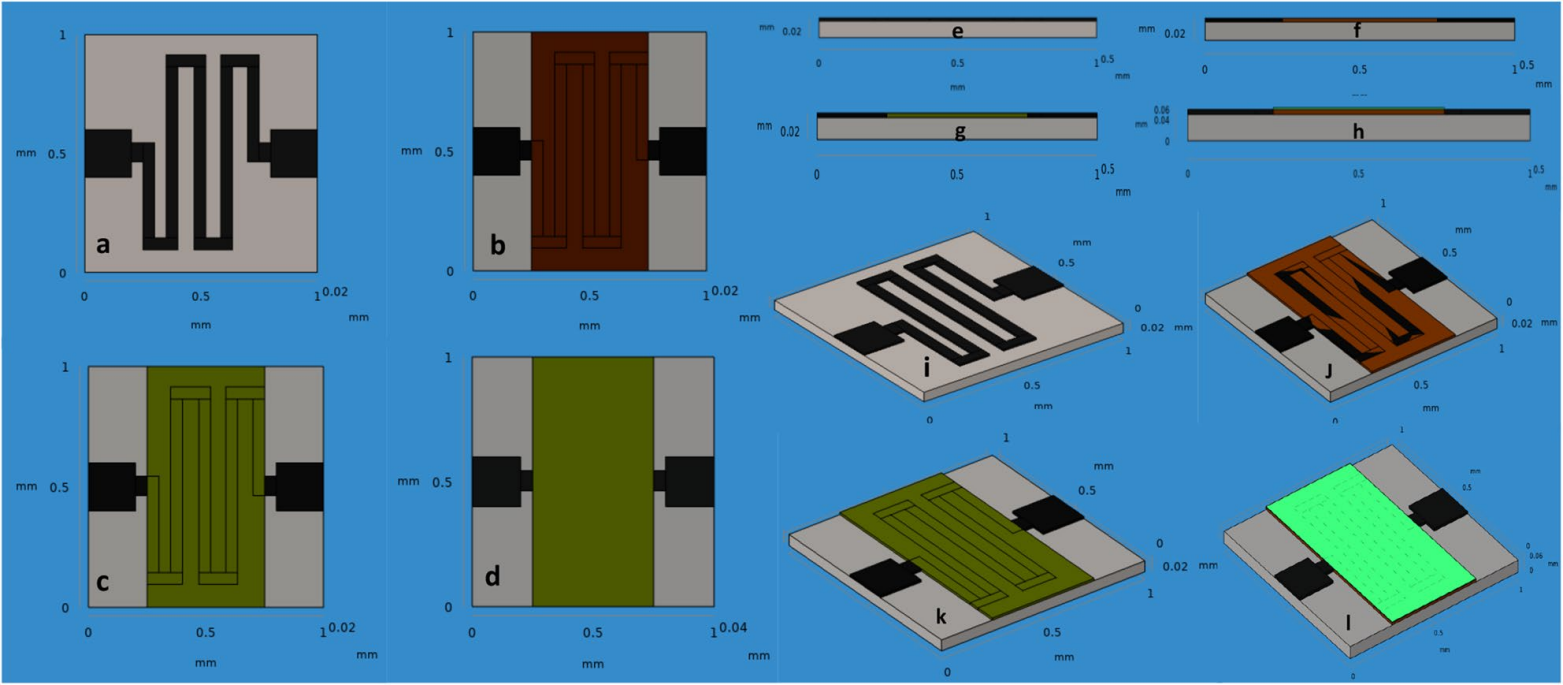
Design of chemical gas sensors (**a**) Sn thin film with Au contact (**b**) Sn–Pd with Au contact (**c**) Sn–rGO with Au contact (**d**) Sn–Pd–rGO with Au contact (**e**–**h**) Cross sectional view of all four sensors and (**i**–**l**) Diagonal view with layers of all four sensors.

Figure 3 presents a schematic designing of the chemical gas sensor device top and cross-section view. The chemical gas sensor with IDE and contacts pads are thick black (gold) on Sn thin film. The Sn thin film act as a substrate and its thickness (0.05 mm) and area is 1 × 1 mm. The pd/rGO hetero-structure was deposited.

The fabrication the process flow is to define the sensor geometry. Then Au metal contacts were patterned. For comparison, the gas sensor is designed with/without active layer deposition. The designed chemical gas sensor has a geometry of the area 1 × 1 mm as represented in Fig. 3.

### Sensing mechanism

Various temperatures, electrical potential, current sensing have been observed based on nitrogen oxides molecules adsorbed on the surface of gas sensor and electrons mobility. Pt/AlGaN heterostructure field effect transistor (HFET) based NH_3_ gas sensor developed and investigated the sensing mechanism. Experimental results through gas sensing that varies the maximal transconductance and threshold voltage are 16.63 mS/mm and 318.1 mV at concentration of 10,000 ppm of NH_3_ gas. Also, its gas response are 113.4 and 2.1 × 10^3^ at concentration of 10,000 and 25 ppm of NH_3_ gas. The resultant from Pt/AlGaN shows that this is the promising material for sensing the ammonia gas^10^. In this paper, they have reported that tungsten oxide based MEMS sensor to respond to nitrogen dioxide at low level of ambient oxygen. It was defined that not only resistive type gas sensor can give high sensitivity but also can operate in low oxygen level at operating temperature of 350 °C. The response was too sensitive to humidity and also have cross sensitivity towards CO, hydrogen at high ppm level. Therefore, it was found that tungsten oxide could be used even in harsh environment and often used in exhaust gases from combustion systems^11^. GaN nanowires and GaN-core/WO_3_ were synthesized using thermal evaporation technique from GaN powder precursor. WO_3_ was deposited on the GaN nano wire using sputtering method. The fabricated gas sensor were analyzed gas sensing response for various concentration of NO_2_ gas are 1–5 ppm. The results shows high response for NO_2_ gas are 125%, 140%, 145%, 159% and 183%. There response were compared with other MOS base gas sensor and this nanowire with WO_3_ thin film gives better response^12^. According to the following equation,

1 $${\text{NO}}_{2} \left( {{\text{gas}}} \right) + {\text{e}}^{ - } {\text{NO}}_{2}^{ - } \left( {{\text{ads}}} \right)$$

2 $${\text{NO}}_{2}^{ - } \left( {{\text{ads}}} \right) + {\text{O}}^{ - } \left( {{\text{ads}}} \right) + 2{\text{e}}^{ - } {\text{NO}}^{ - } \left( {{\text{gas}}} \right) + 2{\text{O}}^{2 - } \left( {{\text{ads}}} \right)$$

On the other hand, the surface states would be altered by the polar Nitrogen oxides molecule, which would manipulate the 2DEG concentration. Therefore, the surface potential of the SnO_2_–Pd/rGO is changed, resulting in the variation of the current of the chemical gas sensor. Electrical potential can mathematically be represented by the Helmholtz model.

3 $$OV = \frac{NsP(\cos \theta )}{{\varepsilon \varepsilon_{o} }}$$

where P is dipole moment between ions, Ns is the dipole density per unit area of the substrate, θ is the angle between the dipole and the substrate surface. ε is the relative permittivity of the active material and εo is the permittivity of free space between layers. Surface potential is major affected by the value of P/ε of the polar molecules.

### Transition metal

Sn-transition material that we use to design a chemical gas sensor i.e.) Tin dioxide with different active materials and their parameters are mentioned in Table 1. Transition metal is select based on the withstand capacity even at low and high temperature. The geometry parameter of the chemical gas sensors are 1 × 1 mm in length x width and depth/height are shown in Table 1.

**Table 1:** Dimension of the materials.

Parameter	SnO_2_	Au	Palladium	rGO
Length (mm)	1	–	0.5	0.5
Width (mm)	1	–	1	1
Thickness (mm)	0.05	0.01	0.01	0.03

**Figure 4:**
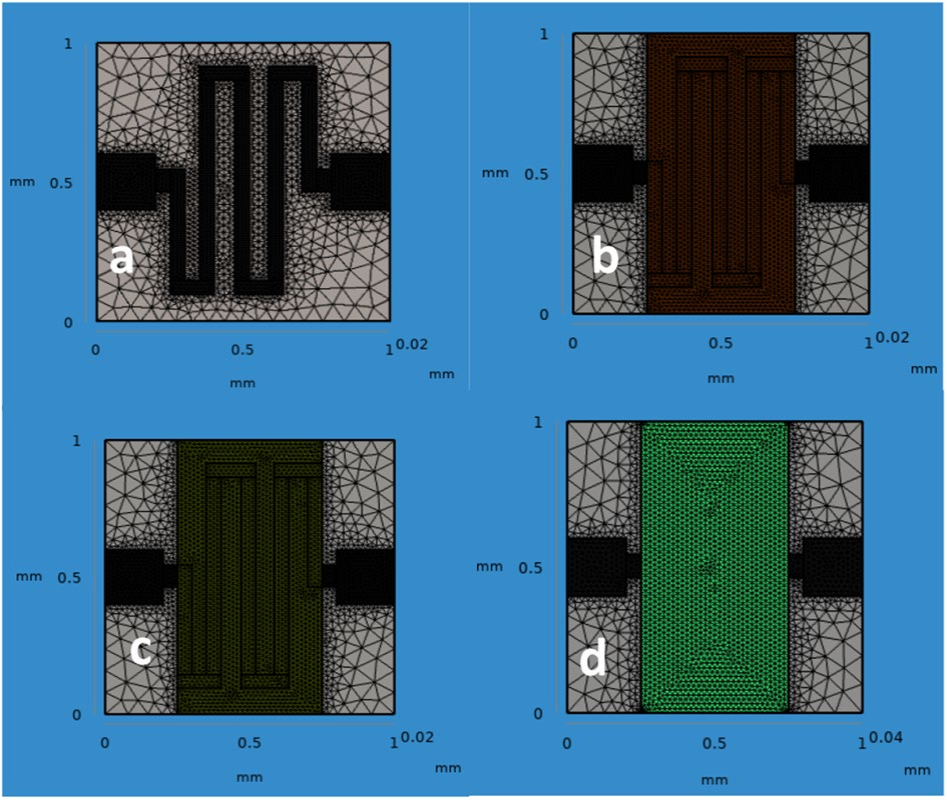
Normal meshing of the chemical gas sensor (**a**) SnO_2_ (**b**) SnO_2_–Pd (**c**) SnO_2_–rGO and (**d**) SnO_2_–Pd/rGO.

The properties of materials used in this research work are tabulated in Table 2. A chemical gas sensor geometry covering area of 1 mm × 1 mm with minimum thickness has been modelled as shown in Fig. 3. Dimension and material properties of the chemical gas sensor used in this simulation are mentioned from Tabulation I and II. Metal oxide gas sensor usually works based on the principle of properties of target gas molecules absorption on the surface of the gas sensor and changes its resistance as the function of various gas concentration. The sensitivity and response of the gas sensor is due to exact active material and input temperature of the sensor.. Hence in this paper, they have designed and simulated the micro heater based gas sensor and their aim to achieve the temperature linearity. They used electro-thermal physics in COMSOL to simulate such micro heater based MEMS gas sensor. Also the micro heater should require low power consumption and better temperature linearity/ uniformity. They optimized the sensor structure single mender, double mender, fan shape, square shape and grill shape of 100 × 100 µm. For the same applied voltage the square shape sensor gives maximum temperature of best result with 99.51%^13^. The chemical gas sensor is designed using the analytical module in the physics of solid mechanics, electrostatics, and heat transfer in solids. In order to attain the required results that are available when we use the FEM method. Normal meshes are customized as its produce uses control over its shape and size on active layers. 3D normal meshing is shown in Fig. 4 (see Supplementary information Fig. S1). The appropriate meshing is applied to build on the solid model using normal mesh structure Fig. 4, a necessary part of the structure has to mesh so as to increase the computational load. In this paper, they have design and simulated the cantilever based MEMS sensor to detect specific molecules in the filed of clinical diagnosis. Drug screening and pathogen detection. Various specific molecules detection are available in software as a physics such as piezoresisitive, optical and piezoelectric device. Among all other physics, piezoresistive type detection become prominent due to the better understanding of the design and results in terms of sensitivity, displacement and changes in resistance. Here Si and SiO_2_ materials chosen as a substrate and various shapes of hole is kept at sensing area. The sensitivity of the microcantilever sensor shows increasing by changing the hole shape, thickness of the material and position^14^.

**Table 2:** Properties of materials.

Parameters	SnO_2_	Palladium	rGO	Gold
Young’s modulus	50 MPa	124 MPa	27.6 MPa	79 GPa
Poisson Ratio	0.36	0.395	0.23	0.44
Density (Kg/m^3^)	7120	12.1	1910	19,300
Thermal conductivity (W/w-k)	66.8	77.8	3250	317
Heat capacity at constant pressure (J/Kg-k)	52.6	250	120	129
Electrical conductivity (nΩm)	115	105.4	103.3	4.56

In this simulation (0.6–4.8) Pa load is applied on the surface of the chemical gas sensor. The surface stress induces potential, temperature and sensible enthalpy characteristics were studied numerically.

To optimize the gas sensor design, dynamic structural analysis was performed to modify the active layers of the chemical gas sensor using FEM structural analysis. Recently, has been focused on the MOS sensors SnO_2_, ZnO, WO_3._ In this paper, SnO_2_ nanorods were deposited with two different material are palladium and ZnO by three different techniques. Firstly SnO_2_ nanorods were synthesized using sol–gel method and then by thermal evaporation method both palladium and ZnO were deposited. The multiple materiasl on single sensor increasing its sensitivity i.e., palladium and ZnO on single sensor gives higher sensitivity and shorter response time at 1–5 ppm concentration of nitrogen dioxide. Hence SnO_2_ nanoroads deposited Pd and ZnO sensor improves NO_2_ sensing than other materials NO_2_ active material for sensor^15^. In this work, they obtained improved device to differentiate the exhaust gases using a standard ZrO_2_ based potentiometer setup. Different voltage is given to sensor to define the parameter which depends upon of individual gas and its concentration. This sensor is developed to sense NO at low level concentration. Sensitivity and response over NO from exhaust gas and its compared with other investigated gas like NH_3_, H_2_ and the mixture of hydrocarbons. Hence this techniques works well and gives better response and high gas sensing towards NO by using a standard zirconia based potentiometric lambda probe^16^. ZnO based sputtered thin film were studied in this paper. Here they kept the film thickness of about 140 nm to sense only NO_x_ and not cross sensing towards mainly CO and H_2_. The maximum NO_x_ sensitivity was observed at 0.67 Pa. Also showed greater sensitivity and selectivity to NO_x_ after adding rare earth materials like Er. This Er doped ZnO film shows high selectivity and quick response time to NO_2_ even at higher temperature above 200 °C and almost sensitivity were linear^17^. Especially SnO_2_ is observed as the best material for NO_x_ sensing mechanism due to its huge selectivity even at low concentration NO_x_ gas molecules. The tungsten oxide based gas sensor has high sensitivity to NO and NO_2_. The sensor response obtained due to the changes in resistance in presence of target gas in air. The fabricated sensor yields very high sensitivity 31 at 200 ppm concentration of NO and 97 at 80 ppm NO_2_ in 300 °C^18^. SnO_2_ is a wide band-gap n-type semiconductor and sensing characteristics of the sensor lies between changes in film resistance resulting from chemisorption and catalytic reaction of gas molecules species with the film layer. SnO_2_ thin films were used initially for detecting NO_x_ & CO. Nowadays, SnO_2_ thin films activated by noble metals (Pd, Pt, and Au) have been found to be more sensitivity, selectivity is shown to give a fast response to NO_x_ gas molecules. The sensing materials of SnO_2_ are promoted with at least 1 wt% metal or metal oxides for NO_x_ detection were analytically observed from the simulation. It was found that some of these metal or metal oxide promoters were critical for improving the sensitivity and selectivity. In this paper, the chemical gas sensors based tin oxide thin films and deposited with/without active materials of palladium, reduced graphene oxide were deposited by a newly developed technique. We described the geometrical and solid properties of the SnO_2_ thin films designed as well as a condition required to fabricate high sensitivity with/without active layer deposited on SnO_2_ thin films. All four different with/without chemical gas sensor was simulated and analytical results tabulated. Especially, we describe with active layers in the SnO_2_–Au and without active layers in the SnO_2_–Au thin films prepared and sensing enthalpy properties of the SnO_2_–Au, SnO_2_–Au–Pd, SnO_2_–Au–rGO and SnO_2_–Au–Pd/rGO for NO_x_ gas molecules in Fig. 3.

## Improvement of sensing performance including the temperature

Tin oxide (SnO_2_) structure and its oxidation state of transition metal are stable and it has vast n-type material with a extensive bandgap of 3.7 eV at 300 K. It displays electronic conductivity at low temperature (even at room temperature) which is great advantages compared to TiO_2_ and high-temperature semiconducting devices. SnO_2_ is instigated by fast adsorption between H_2_O and adsorption oxygen species (O_2_, O^−^, etc.) & in return delivering the adsorbed O_2_ ions and free electrons, water (H_2_O) is adsorbed on the surface of the oxides layer in particles and –OH forms, then the chemical reaction takes place, that was detected by using electronic devices. It detects the fierce absorbent between water and oxygen molecules to increase the conductivity. Tin oxide gives several unique optical and electronic properties: receiving differs in an air environment. Tin oxide element has been popularly accepted in multiple chemical sensors and carriers transferring terminal such as gas sensors (pollutant oxides likes CO, HC, NO_x_, PbO_2_). Many researchers have paid a huge amount of attraction to SnO_2_ as a gas sensing devices. Although, only a minimum papers and few have look over tin dioxide as a humidity sensing metal. In this work, SnO_2_ thin film used as a substrate with a dimension of 1mmx1mm square shape chemical gas sensor. Because tin dioxide belongs to the transition conducting metal. Already we discussed its general properties, such as dimension and parameter data. We discussed the new technique used for designing and also its dimension & parameter which are basically used for the application such as electronic devices, transparent electrodes and sensors and its mainly focused on the material conductivity, ions mobility and ability to form p-type conductivity.

**Figure 5:**
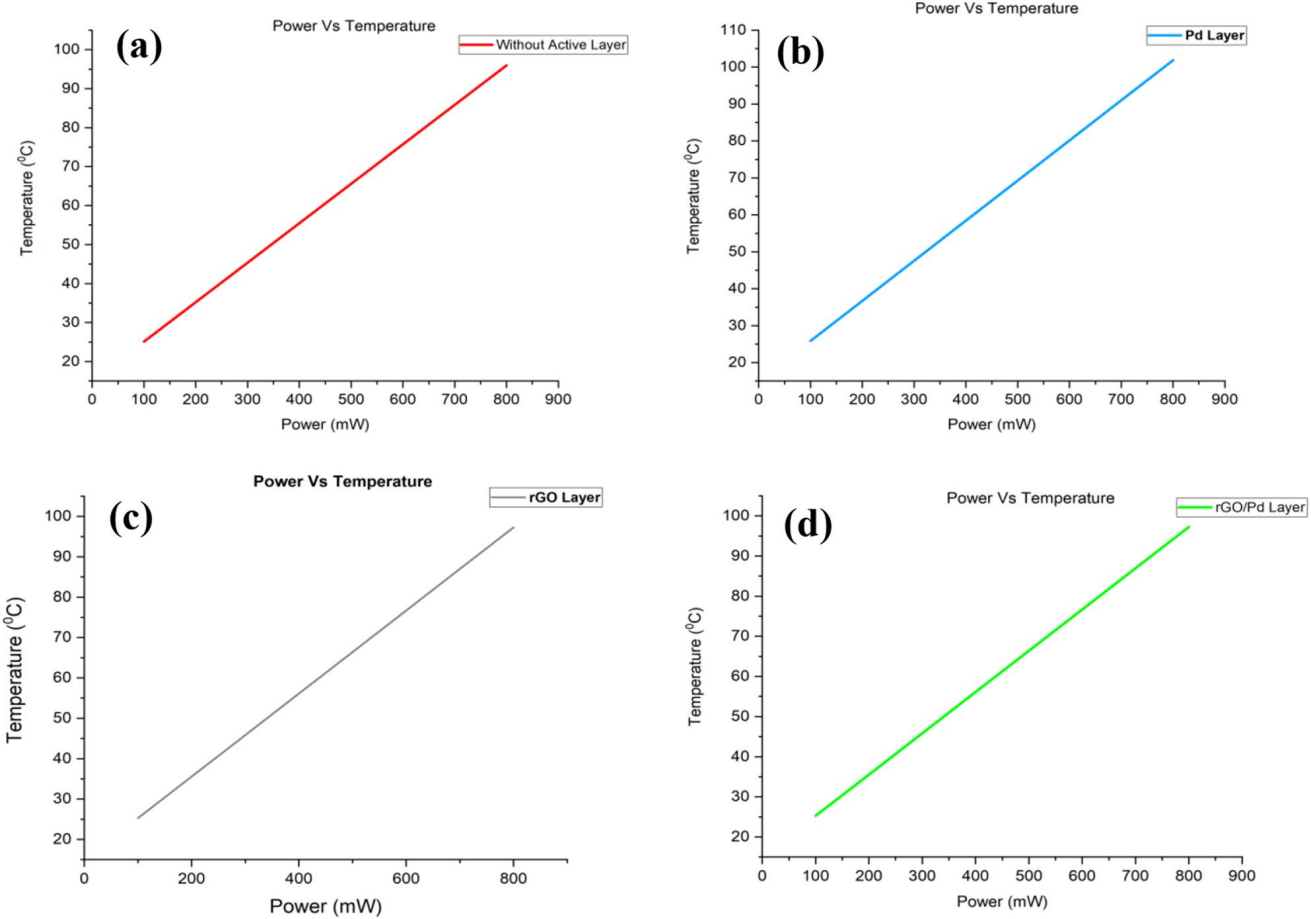
The operating temperature versus applied power curves of substrates used in the simulation of chemical gas sensors: (**a**) SnO_2_ thin film (**b**) SnO_2_–Pd (**c**) SnO_2_–rGO and (**d**) SnO_2_–Pd/rGO.

In order of merits to improve the reaction towards nitrogen oxides, we are preferred to use sensing materials that are used to sense target gas molecules. Therefore, Pd, rGO active materials used as a sensing material, and it's designed as different samples which are clearly explained in previous paragraphs. The flexibility of chemical gas sensors is one of the most advance in the modelling of next generating sensing devices and other electronic application devices. In this paper, authors clearly explained about all fabrication techniques especially on the area of printable electrodes on flexible sensor devices. As it is mentioned the sensor device will be flexible in nature it also has it own merits and demerits. The main crucial area in this flexible device was fabrication due to its soft flexible, Cost or micro level fracture while developing. Here they have discussed about the two main fabrication methods that is Top and Bottom. The selections methods based on material and its physical properties. Also they studied about challenges due to cost, synthesis method, ink and production^19^. This paper reports the electrodeposition of polmers in (LiNi0.5Mn1.5O_4_) porous lithium nickel manganese oxide by cyclic voltammetry process. Here polymer were coated on LNMO and its acted as a cathode during electrochemical impedance analysis. It improves capacity and operating voltage. This is due to the filling of polymer over the porous of LNMO, that increases its working efficiency. Therefore, in this paper they have successfully fabricated a microbatteries by LNMO as a cathode, polymer as a electrolyte and TiO_2_ nanotube as a anode^20^. Innovative multi functioning shoes were developed and discussed in this paper. The main parts of the shoes are divided into two column, they are rubber material as a heel to play as cushioning and damping and other one is electric circuit column to do energy harvest, storage, charging during walking movement. The working this developed rubber shoe were tested multiple times and come up with optimized structure human weigh and performance. Therefore, stress and energy harvesting were tested and analyzed in different environmental condition. From the results it was found that the rubber multi functioning shoes provides more stability, versatility under different condition^21^. Flexible micro battery were developed for an eye tracer. Also discussed about how to develop micro level and embed the circuit into the lens such as ASIC and photodiode. The developed ring like charging device has a area of 0.75cm^2^ and capacity of 43 µAh cm^−2^. Simulated design yields 0.35 µm power consumption by allowing power for 3 min^22^. Gas sensors plays very important role in current science and our daily life. Nowadays its become very intensively, how to improve the performance and efficiency of gas sensors. In this project, they implemented Titanium dioxide based sensor for cyber chemical sensor to medical diagnosis. Titanium dioxie physical and chemical characteristics were studied for the development of gas sensor. The main focus on this study is real time monitoring and diagnosing. The importance of this developments on fabrication of porous titanium dioxide chemical gas sensors and its results shows the convenient and feasibility and provides the security and perform the diagnosis. Titanium dioxide has increasing its sensitivity performance and it has unique properties on sensing material^23^. Due to this valuable reason our following studies: we obtained from gas Sensor using SnO_2_ substrate thin film. Since SnO_2_ thin film can be used till 500 °C, the electrodes can withstand with the substrate at 500 °C. In addition to the metal oxide nanomaterials can be treated up to 500 °C. The resultant graphs between temperature deviation versus the applied power, current and sensible enthalpy. By verifying the optimum active materials which give more selectivity towards target gas molecules. However, in this work higher operating temperature is 100 °C leads to the stress of the tin oxide substrate. The temperature deviation of the electrode is depends upon the applied power, current, and sensible enthalpy. In this case, applied power was changed from (100–800) mW to evaluate the gas sensor to operate from 25 °C to 95 °C temperature is shown in Fig. 5

## Simulated results and discussion

The manufacturing of chemical gas sensors based on nanocomposite materials and creating hetero-junction provides a great opportunity to increase the reaction properties of oxides layer. The conjunction of these nano active materials and hetero-junction with different active layers gives electrical conductivity resulting in charge mobility between hetero-junction.

Reduced graphene and ZnO nano materials were used for the detection of voltaic organic compound identification. The sensing performance were carried out by experiencing with various level of C_2_H_5_OH and C_3_H_6_O. The developed rGO sensor shows greater in sensing response compared to ZnO material. From the observed results is showed that this is the promising material for sensing the environmental pollutant. Fabricated sensor can also used for breathe analysis^24^. In this work, for the first time NiO–ZnO 1D nanowire were fabricated using vapour liquid solid mechanism. Also the study involves in characterization of morphology like shape size. Therefore it is heterostructure. From the morphology analysis it is identified that ZnO nanowire is completely covered with NiO nanowire. The diameter of the samples were varied from 15 to 60 nm. Through XRD analysis, orientation of the element were found ZnO is 101 and NiO is 200 crystallographic plane. The gas sensing response were observed and compared. Therefore, NiO–ZnO fabricated sensor shows greater sensing characteristics compared with NiO sensor towards voltaic organic components^25^. CuO–TiO_2_ were synthesized and deposited on Al_2_O_3_ by chemical vapour deposition. Based on the growth during synthesis process and deposition technique, sensing characteristics were improved to O_2_ and H_2_ atmospheric gas. Another sample is added with active material gold by sputtering to increase its functionality on sensing. Synthesized sample were characterized by XRD, SEM, XPS,AFM and SIMS. Also they have done testing on CuO–NiO and CuO–NiO–Au and found out that the fabricated sensor work as a resistive type even at low working temperature. They highlighted that after adding gold into the CuO–NiO material, considerably improved the sensing performance^26^. This unique effect is purely due to the active materials high-performance chemical gas sensors based on nanocomposites. SnO_2_–Pd/rGO type gas sensor given output values due to the oxidizing and reducing gas molecules up to 100 °C. The peak gas sensing reaction was reached due to the deposition of the interfacial area between the thin film SnO_2_ and active material Pd/rGO to increase the duration of the charge carrier.

**Figure 6:**
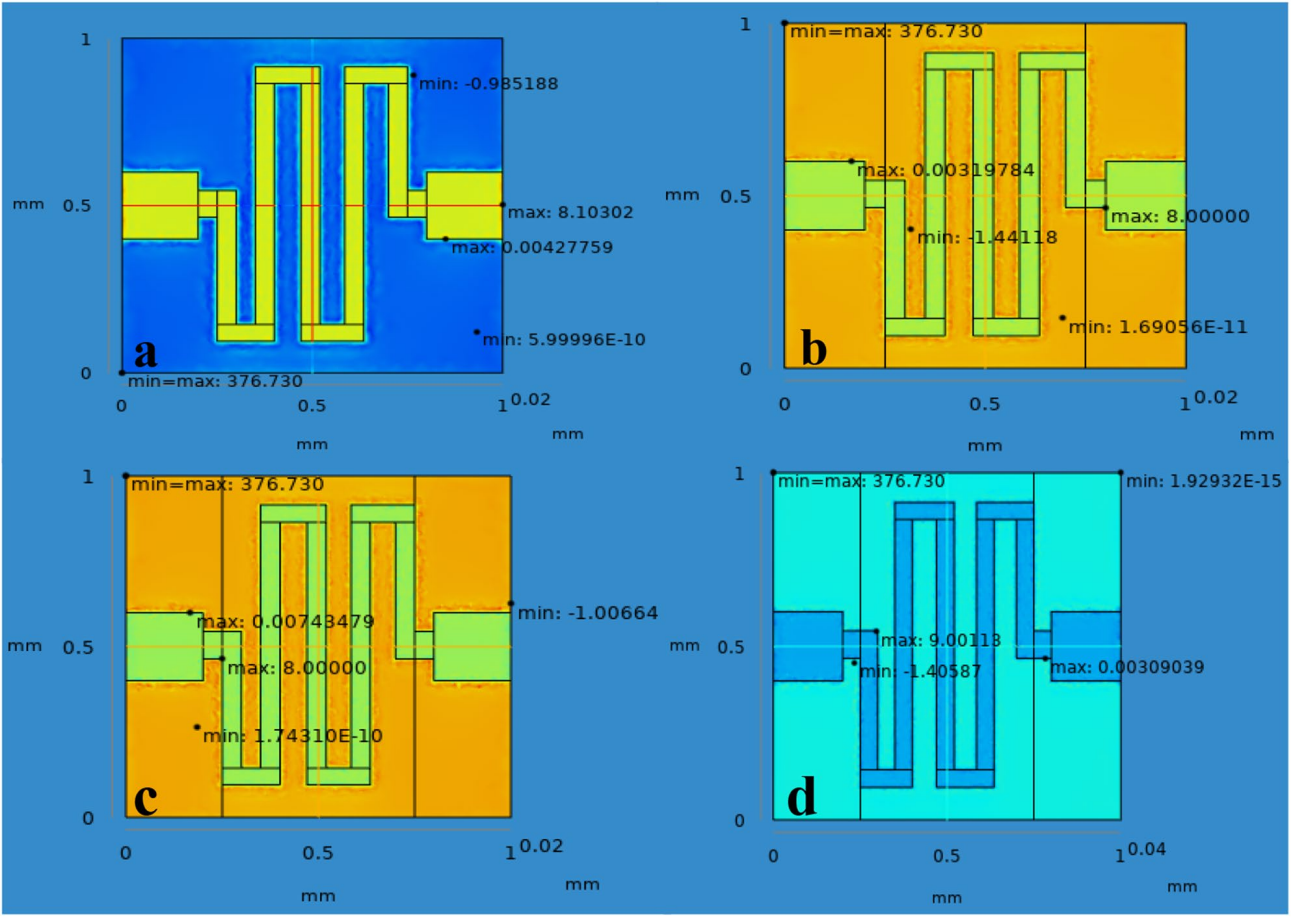
Current density across the chemical gas sensors: (**a**) SnO_2_ (**b**) SnO_2_–Pd (**c**) SnO_2_–rGO and (**d**) SnO_2_–Pd/rGO.

**Figure 7:**
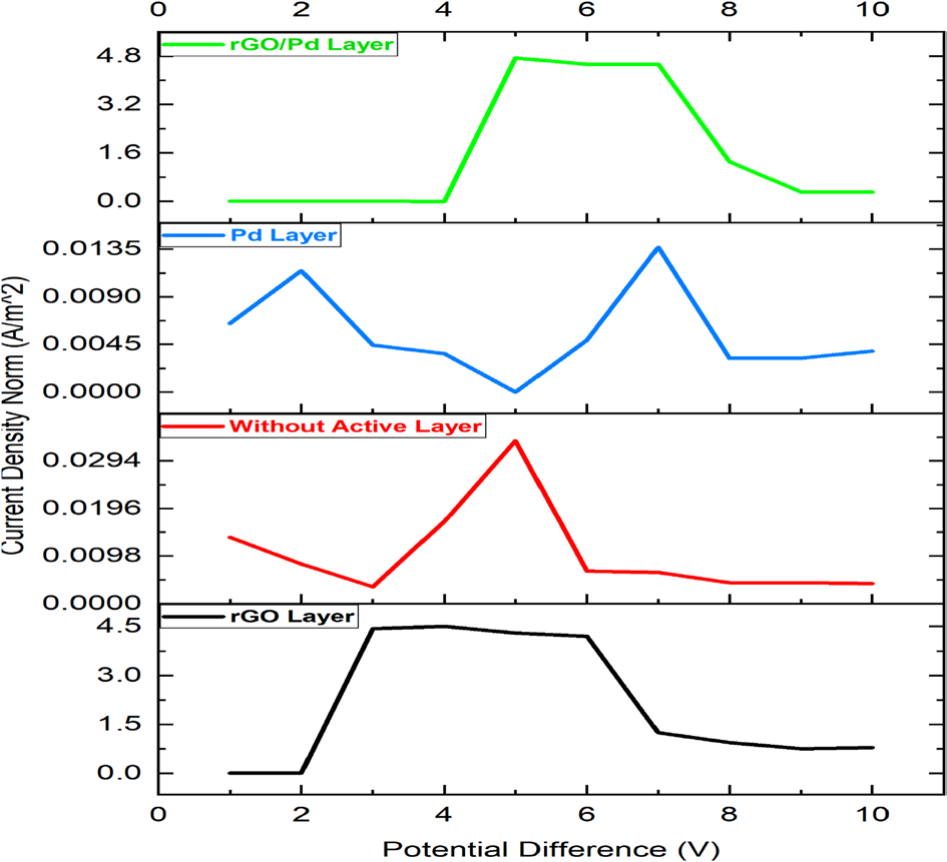
V–I response of Chemical gas sensor with and without active layers i.e., SnO_2_ thin film, SnO_2_–Pd, SnO_2_–rGO & SnO_2_–Pd/rGO.

**Figure 8:**
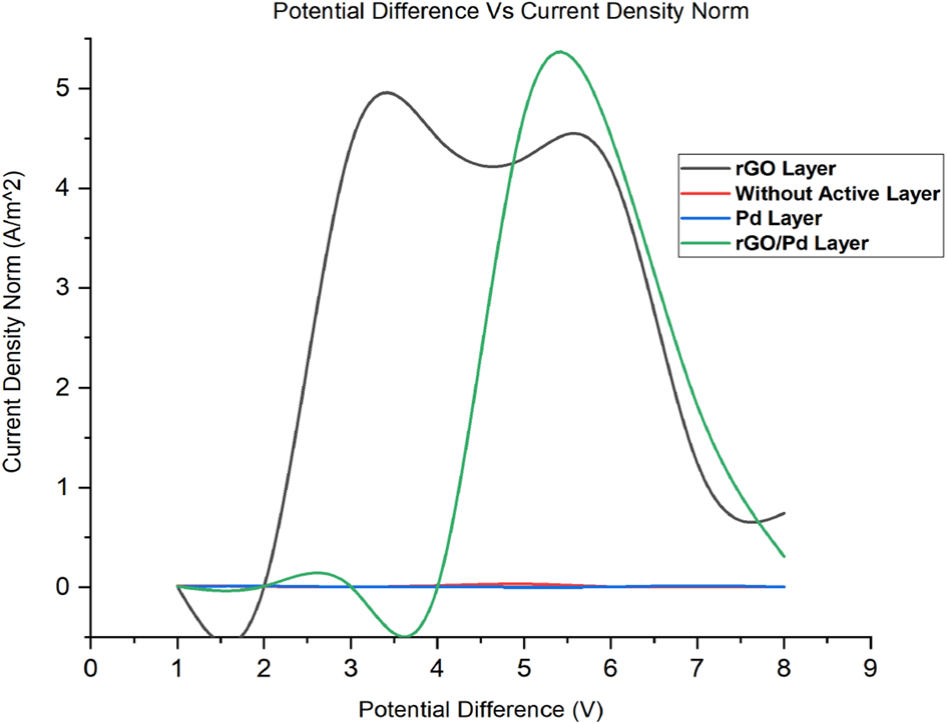
Effect and comparison of V–I response of all four chemical gas sensor i.e., SnO_2_ thin film, SnO_2_–Pd, SnO_2_–rGO & SnO_2_–Pd/rGO.

**Figure 9:**
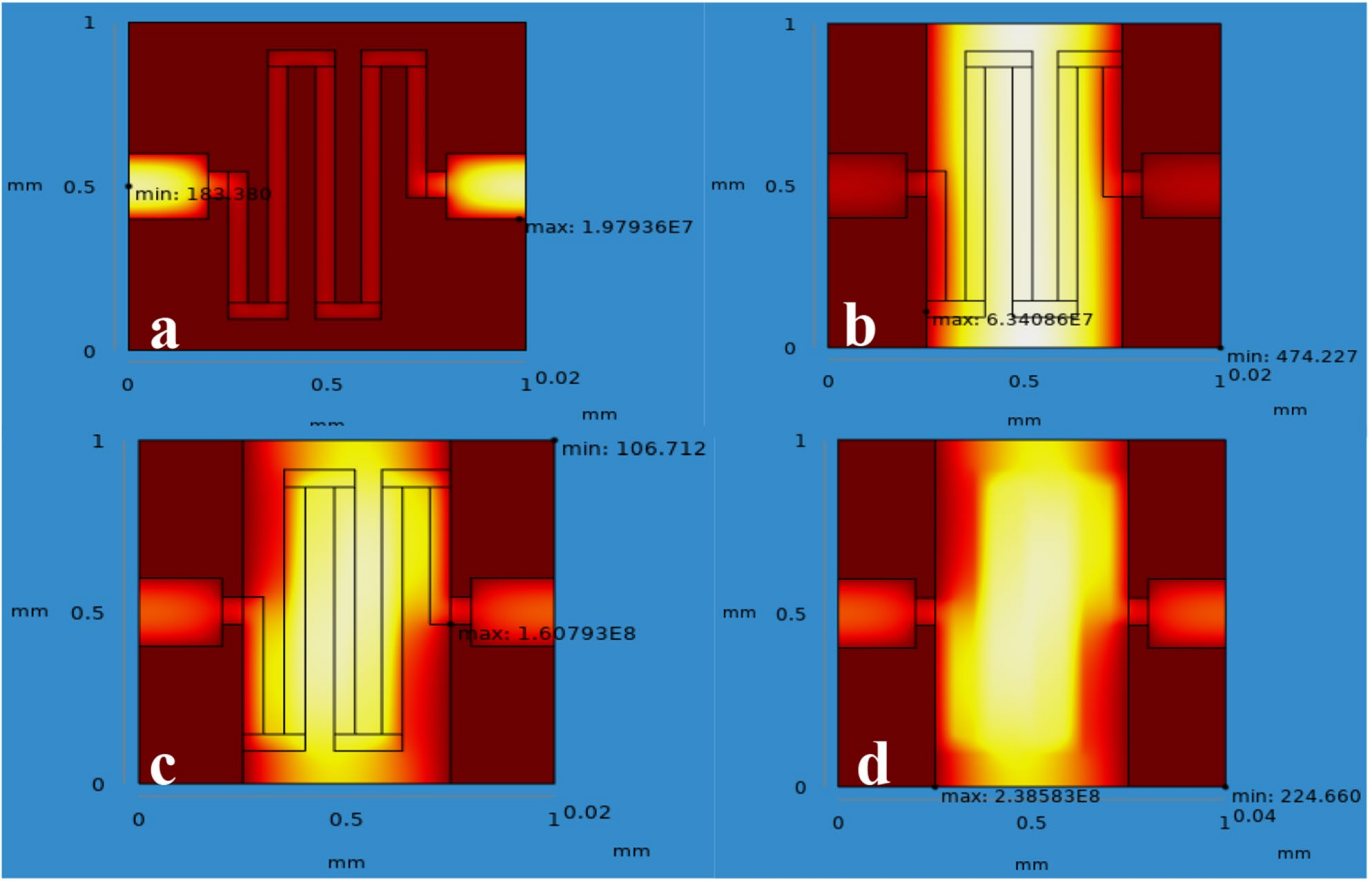
Temperature distribution on sensing layer of the chemical gas sensor: (**a**) SnO_2_, (**b**) SnO_2_–Pd, (**c**) SnO_2_–rGO and (**d**) SnO_2_–Pd/rGO.

**Figure 10:**
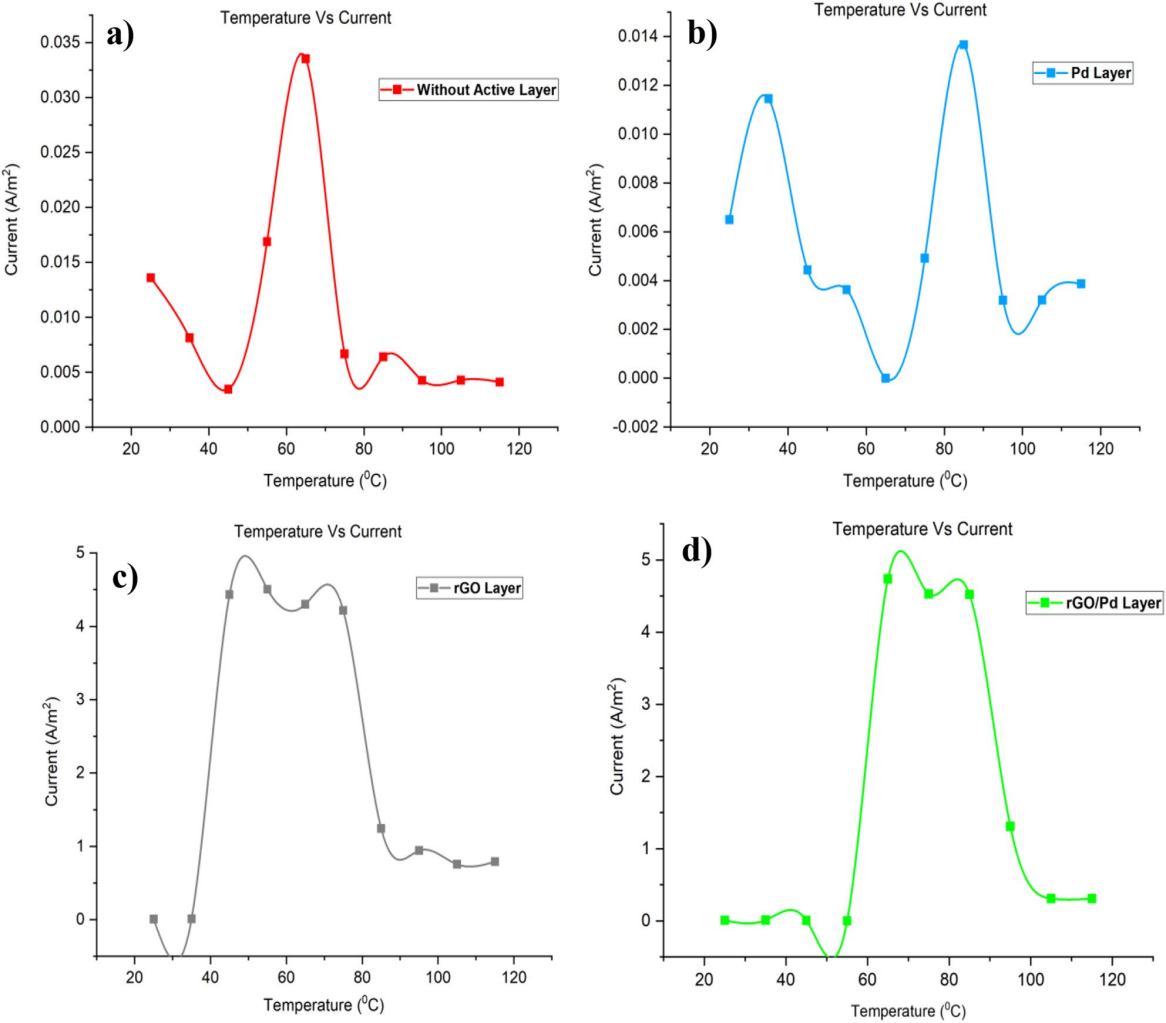
Sensor response towards NO_X_ gas molecules in different working temperature to all four design i.e., (**a**) SnO_2_ thin film, (**b**) SnO_2_–Pd, (**c**) SnO_2_–rGO and (**d**) SnO_2_–Pd/rGO.

From the simulation, the chemical gas sensor behavior in terms of potential, current density, temperature, sensible enthalpy, total displacement, load, pressure, volumetric strain and surface charge density as shown in following figures were obtained by power, voltage and applied force on the surface of the chemical gas sensor. Figure 6 shows the potential distribution on chemical gas sensors and Fig. 7 (see Supplementary Fig. S2) shows a comparison between all four designs of the same applied potential on the sensing layer Vs current density norm A/m^2^. The electrical potential varied from (1–8)V as shown in Fig. 7. The resultant current density norm has a little drop with raising due to temperature and lattice scattering of the 2DEG. The current density norm decreases remarkably between 85 °C and 95 °C. The saturated current only attains for SnO_2_–Pd/rGO and SnO_2_–rGO is 4.8 A/m^2^ and 4.5 A/m^2^. This study indicates the V–I response of the chemical gas sensor up to 100 °C. The effect of all four designs shown in the comparison graph plotted in Fig. 8 and can seen in Supplementary Fig. S3. Chemisorption can happen on the top surface of the gas sensor in the presence of a reactive atmosphere. This reaction totally depends on the condition of temperature, pressure, and power used. These are extremely applied in the case of NO_x_ species that can change the semiconductor surface. The temperature diffusion on the chemical gas sensor are reported clearly in Fig. 9 (Supplementary Fig. S4). However, the gradual response was observed in the sensor Vs different operating temperatures up to 100 °C is shown in Fig. 10

**Figure 11:**
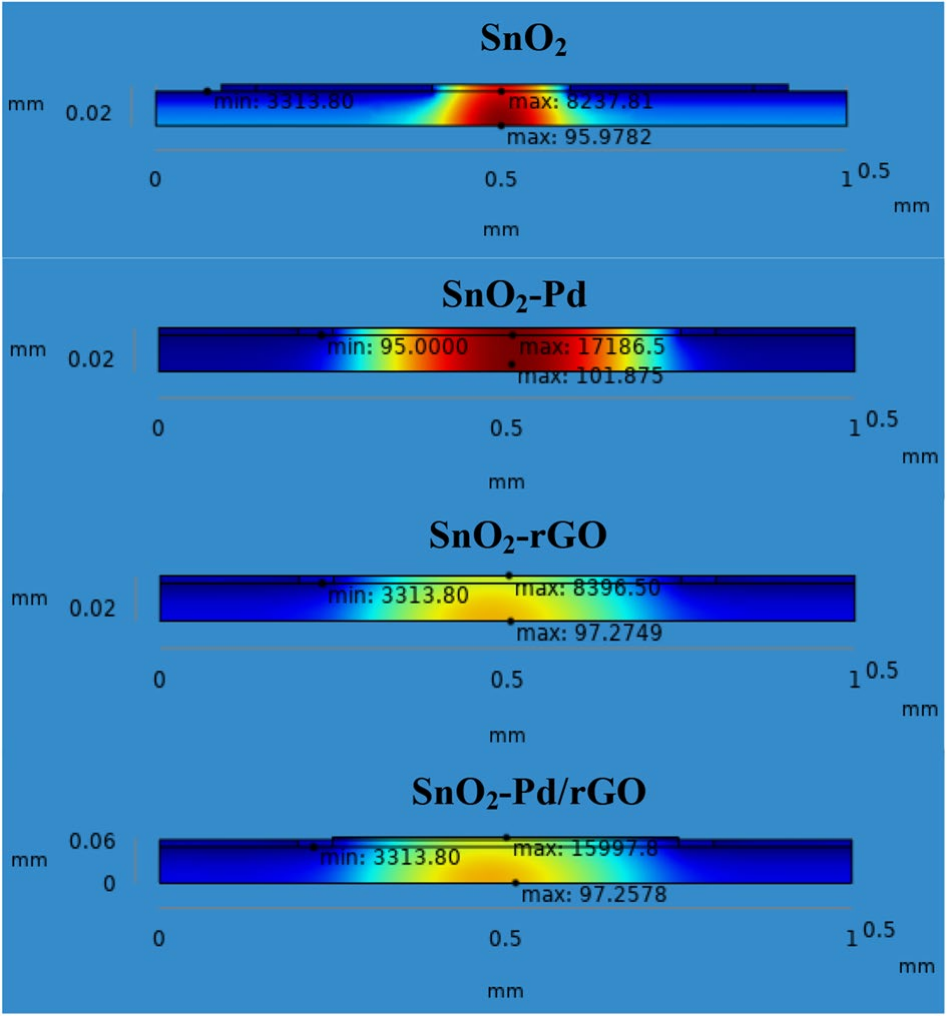
3D Simulation NO_x_ sensing enthalpy flow over thin film chemical gas sensor: (**a**) SnO_2_, (**b**) SnO_2_–Pd, (**c**) SnO_2_–rGO and (**d**) SnO_2_–Pd/rGO.

**Figure 12:**
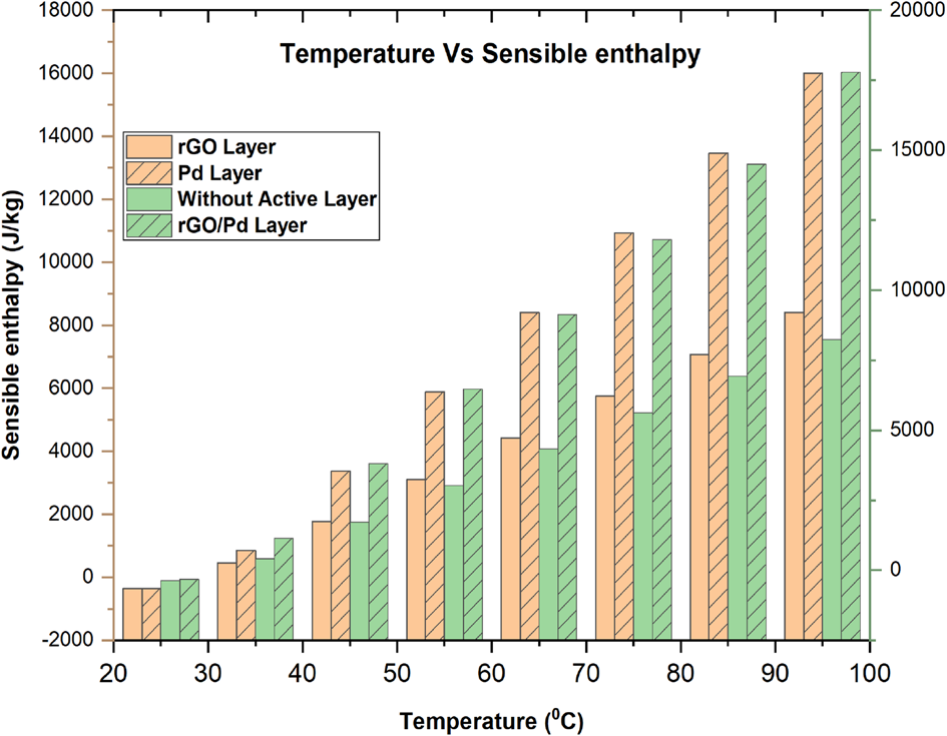
Sensor response of chemical gas sensors with/ without active layer towards NO_x_ gas molecules in different working temperature.

Results from Fig. 11; can be seen in Supplementary Fig. S5 simulation are then used to compute the sensitive behavior such as sensible enthalpy and current changes resultant from the applied temperature. Chemical gas sensor sensible enthalpy is obtained by measuring the changes in sensitivity when a load is applied to the chemical gas sensor with respect to temperature. From the Fig. 12, maximum sensitivity is shown in both SnO_2_–Pd/rGO and Pd active material sensor at most of the operating temperature up to 100 °C. The effect of all four designed active layer gas sensors to improve chemical gas sensor sensitivity is studied by comparing all four active materials.

**Figure 13:**
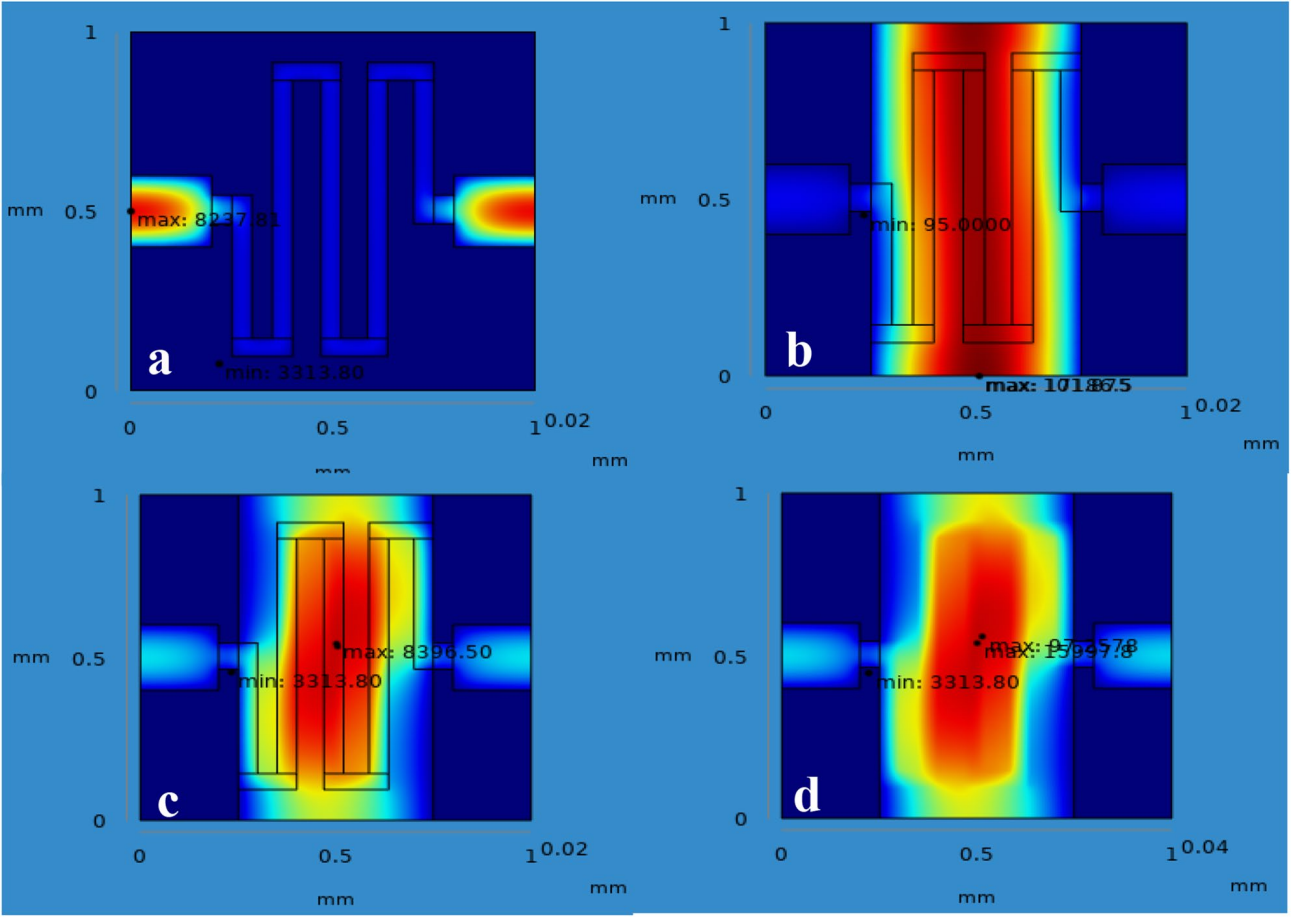
Isothermal contour distribution due to temperature on the thin film chemical gas sensor: (**a**) SnO_2_, (**b**) SnO_2_–Pd, (**c**) SnO_2_–rGO and (**d**) SnO_2_–Pd/rGO.

**Figure 14:**
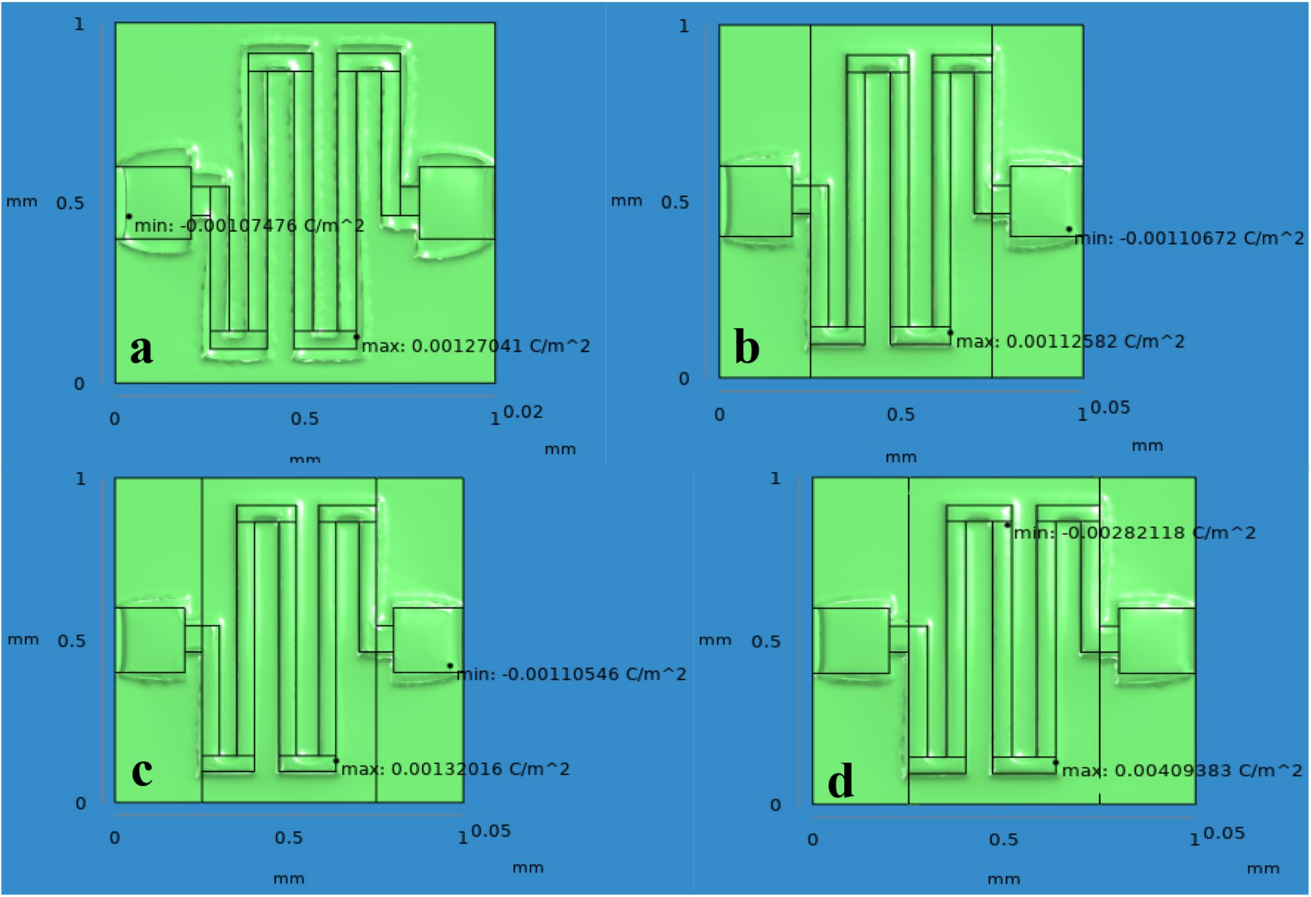
Stress distribution on the chemical gas sensor: (**a**) SnO_2_, (**b**) SnO_2_–Pd, (**c**) SnO_2_–rGO and (**d**) SnO_2_–Pd/rGO.

Meantime the sensing achievement of SnO_2_–Pd/rGO it may be that improved due to depositing the active materials. The effect of Pd/rGO on the sensitivity layer to investigated NO_x_ gas molecules. The high response was obtained from SnO_2_–Pd/rGO at a lower temperature of 100 °C. Gas sensing properties in terms of isothermal contour for different temperature as clearly shown in Fig. 13. The temperature, current, and pressure changes value from minimum to maximum. Sensitivity towards NO_x_ gas molecules increases after active layer materials deposition. As the function of sensitivity towards NO_x_ gas molecules with/without the active layer is improved and observed. However, the sensitivity of chemical gas sensors with active layer shows increasing faster. The position reason is that SnO_2_–Pd/rGO surface is easy for NO_x_ molecular desorption. The consequence of the low temperature studied to own a impact on the performance of the chemical gas sensor was studied as well. The current changes and sensitivity as a function of potential are also plotted in Figs. 8 and 12. The sensing properties are significantly enhanced with increasing temperature/voltage. Micro SnO_2_–Pd/rGO chemical gas sensors have shown great potential and temperature to detect low NO_x_ concentration with fast response time and temperature. Similarly, designed sensors shows the stress distribution on overall area of the substrate for applied load is clearly shown in Fig. 14 (see Supplementary Fig. S6).

**Figure 15:**
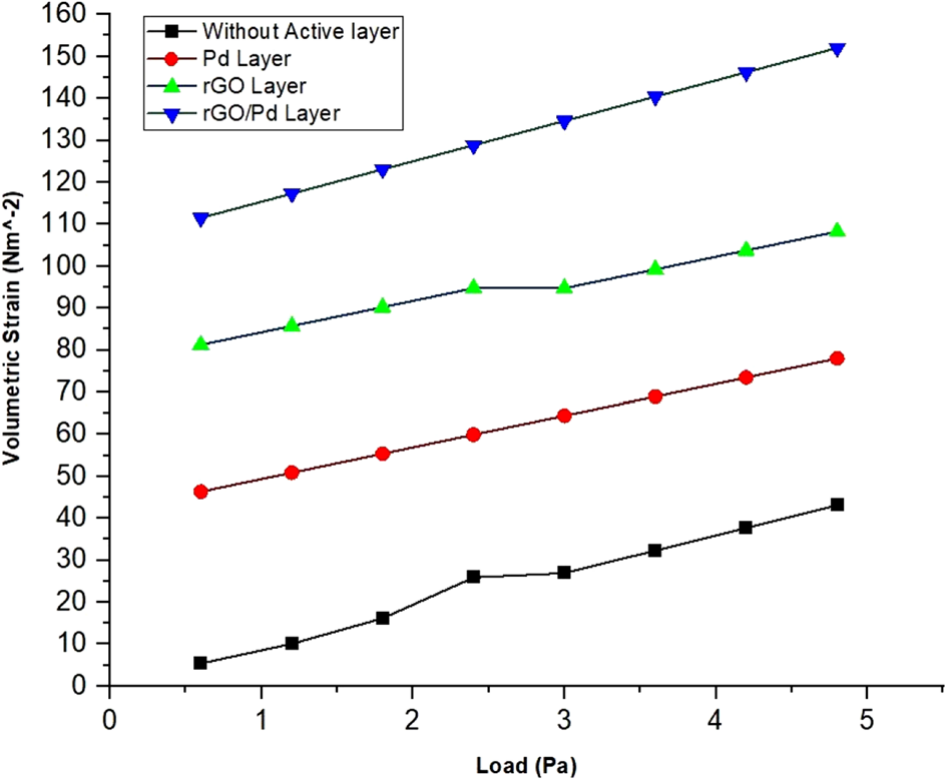
A Volumetric strain comparison of all four design for the same applied load.

**Figure 16:**
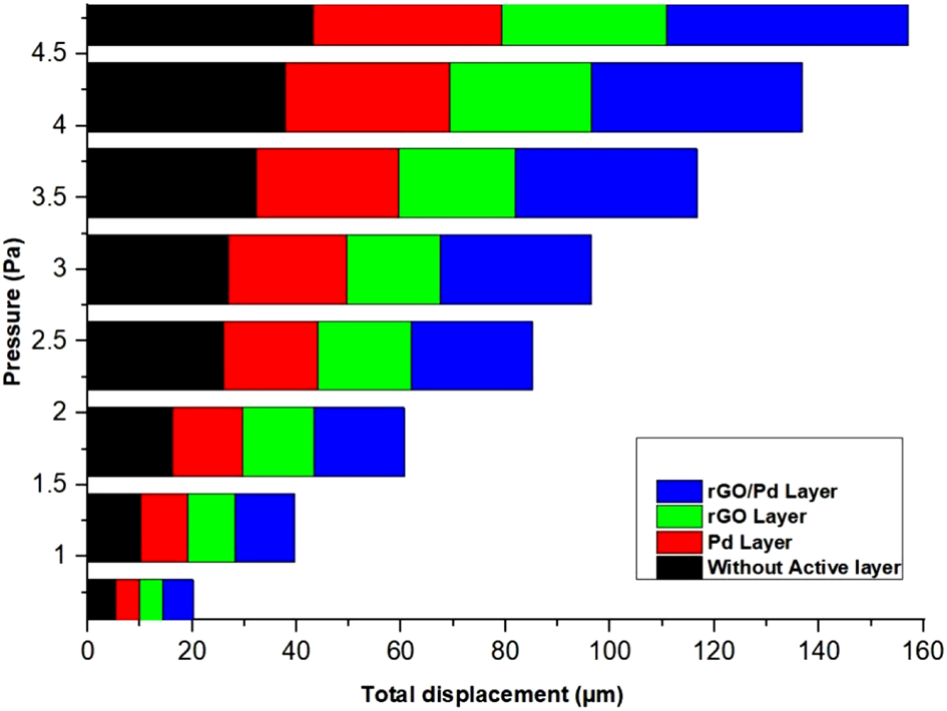
A displacement comparison of all four design for the same applied pressure.

**Figure 17:**
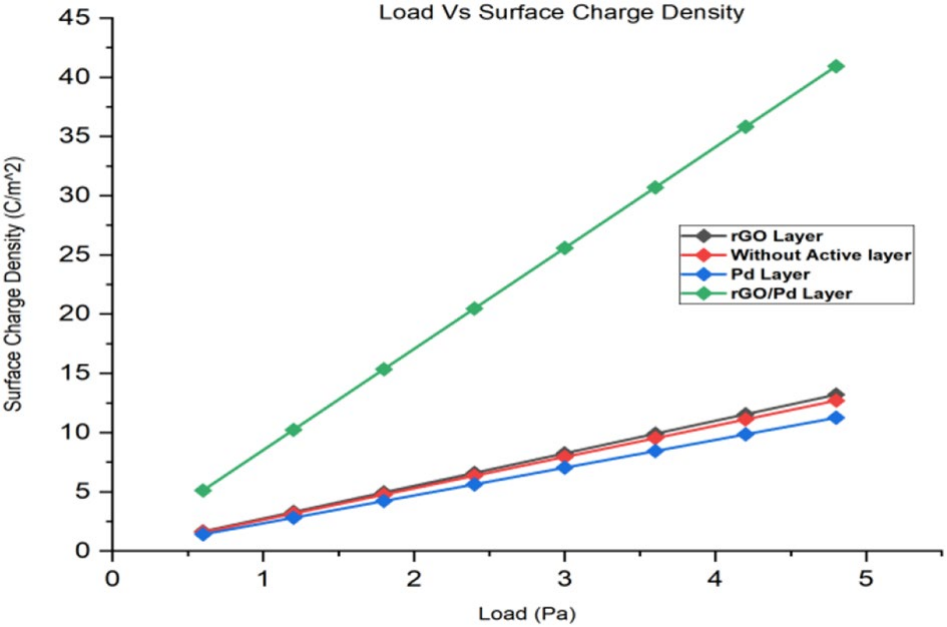
Surface charge density comparison of all four design for the same applied load.

As such SnO_2_–Pd/rGO chemical gas sensor is considered throughout the simulation process where higher displacement & surface sensitivity are also expected. From the graphical representation of volumetric strain for applied load is analyzed and without active layer shows the maximum volumetric strain for applied load is shown in Fig. 15 (see Supplementary Fig. S7). Applied Pressure in terms –of pascal on the surface of the chemical gas sensors gives the respective displacement due to the mass deposited on the layer of the sensors and results were compared for all four designs in Fig. 16. Typical surface charge density of all four design for applied load that illustrates the sensitivity of the chemical gas sensor to NO_x_ gas molecules trapped on the active layer of the nanocomposites of the designed chemical gas sensor is given in Fig. 17 . It's clearly observed from the result that togetherness of Pd/rGO nanocomposites & rGO on the substrate significantly improving the surface charge density value with respect to increasing in load. For the designed chemical gas sensor, temperature, power, sensible enthalpy, etc., are distinguished and elevated sensing performance due to an active layers are clearly shown from above-simulated figures. All these results help us to observe the active layer sensitivity of the designed chemical gas sensor that is used.

## Response of the thin film gas sensor—(Ra/Rg)

**Figure 18:**
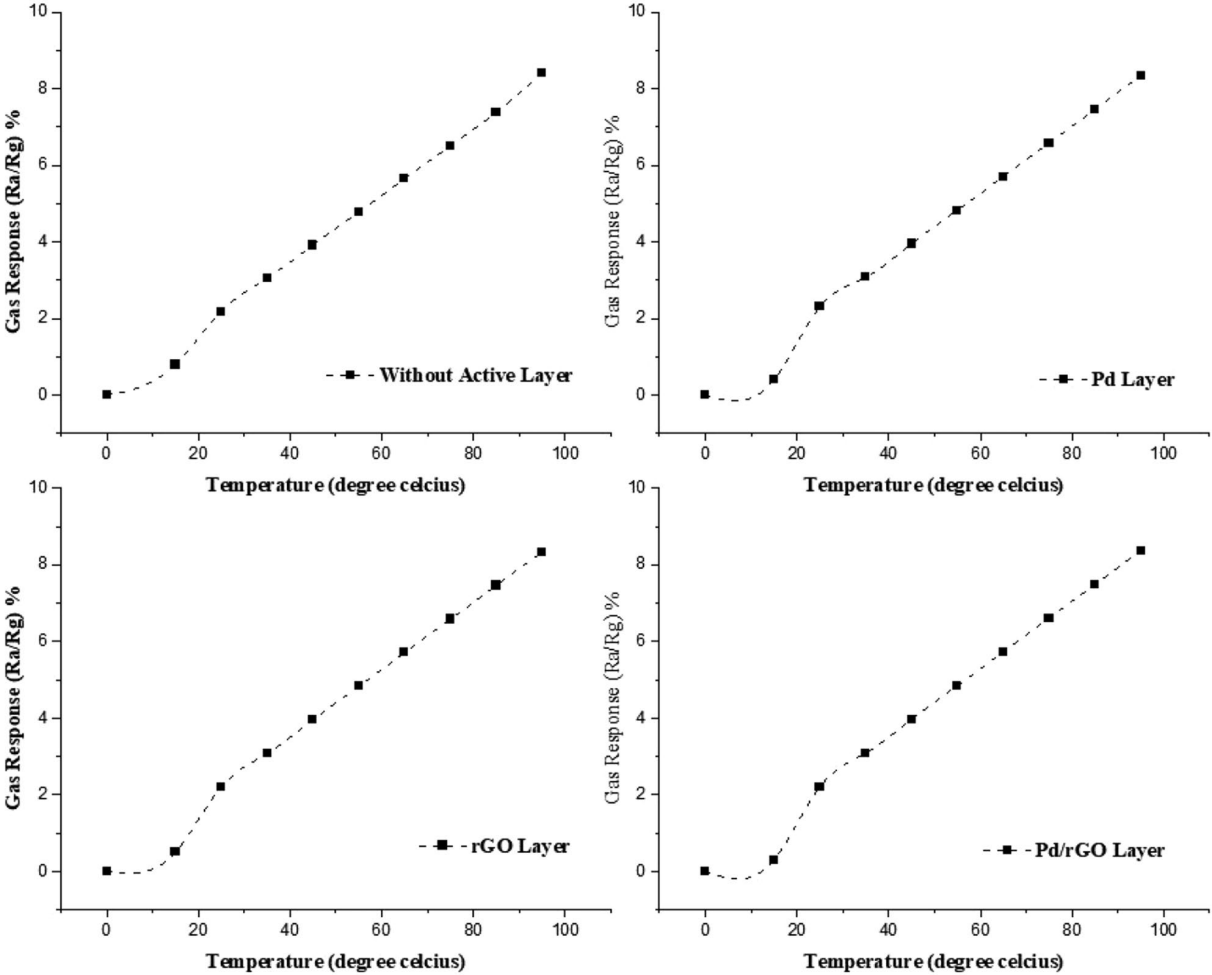
The simulated results of response at various working temperature.

The simulated results show the good concurrence that can be taken into account before going for real-time practical application. Also, the response of the designed sensor was calculated manually by using the following formula in order to find out the response for various load, Temperature. Results were plotted as a graph temperature vs response in Fig. 18. The response of the gas sensor can be characterized as the ration of the sensors resistance in the environmental pollutant air to the resistance in the existence of the target gas molecules, (Ra/Rg) versus with temperature.

4 $$R(T) = R_{0} \exp \left( {\frac{{T_{0} }}{T}} \right)^{m}$$

where T_0_ is characteristics temperature and m = 1/4 corresponding to the 3D-VRH. Therefore, by increasing the operating temperature with load in terms of power will increase the response in percentage. As it is shown in below Fig. 18; results obtained from the designed thin film gas sensor can be seen in Supplementary Fig. S8, at sensing the concentration of NO_x_ i.e.) load, the sensor’s response is getting gradually increased. The principle attributes to increase of the electrons with the number of nitrogen oxides gas molecules, resultant in decreasing sensor resistance with increasing in an operating temperature.

## Computational methods: analytical model

For the simulation of the resistive gas sensors used three major physics, they are solid mechanics, electrostatics and heat transfer in solids. These physics are included in the ordered and simulated in 3D platform. Mainly the simulation involves in three process state and its describes the entire process flow of gas sensing (a) Operating temperature (b) Target gas (c) Humidity and (d) Electrical readout due to the interaction and collision between target chemical species to the active layer.

### Solid mechanics

In this work by using solid mechanic physics, we designed thin film based gas sensor with/without active layer and its parameter and electrical properties clearly mentioned in Tables 1 and 2. Here the active layers are palladium (Pd), reduced graphene oxide (rGO) and double active layer i.e.) reduced graphene oxide on palladium layer. After the sensor is designed successfully, it is important to make into a single device by using union function from boolean expression.

### Electrostatics

When the active layer of the gas device is experiencing with chosen chemical molecules, then it gives resultant in the change in the sensor’s resistivity through electrical readout can easily figure it. Generally, the interaction between the target gas molecules and the active layer changes its resistive value and this can be explained as a basis of Poisson’s equation,

5 $$- \nabla^{2} V = 0$$

Whenever collision happening between target molecules and the active material due to the steady addition of target chemical molecules form of load, then COMSOL tool uses Poisson’s equation to build electrical parameters of the gas sensor such as current, temperature, voltage and other available response.

### Boundary condition

In the most studies, boundary conditions for the simulation of resistive gas sensor are fixed at the base of the gas sensor and flexible at the top layer of the active layer. We followed the same in this work, and the boundary condition has two different domains for solving the heat transfer and electrical equation.

### Heat transfer

Working temperature of the developed device transfers the heat by heat conduction between the substrate and the active layer, then its occurred due to the collision of the gas molecules and electrons mobility in a surface. The Fourier’s law gives the minus disgonal in the temperature is directly proportional to the temperature of the material we chosen, is the generally a law that governs the heat conduction given by

6 $$\overrightarrow {q}^{n} = - k\overrightarrow {\nabla T}$$

where q^n^ is flux density, K is conductivity and T is temperature of the fabricated device. Then the above fourier’s equation given below

7 $$q^{n} = - k\left[ {i\frac{dT}{{dx}} + j\frac{dT}{{dy}} + k\frac{dT}{{dz}}} \right]$$

In the direction of n

8 $$q_{n}^{n} = - k\frac{dT}{{dn}}$$

Therefore, the total heat flux can be rewritten as

9 $$q^{n} = iq_{x}^{n} + iq_{y}^{n} + iq_{z}^{n}$$

### Analytical model of the gas sensor in the COMSOL platform

Modelling and simulation of the gas sensor is depends upon the parameters such as geometry and shape, size, mesh and the chosen materials. This simulation deals with optimization, hence its necessary to verify the parameter and it has to improved to attain the maximum sensor’s response and to successfully achieve the desired resultant. The parameter used in this simulation were showed in figures and its extracted from COMSOL software. The same simulated sensor’s parameter will be used in the future fabricating real sensor. Geometrically, the gas sensor consists of three parts: Tin dioxide substrate, electrode and the active layers.

*SnO*_*2*_* substrate.* The dimensions of the tin dioxide that has been designed in this work is 1 × 1 × 0.05 mm. The substrate parameters and properties have been from the Tables 1 and 2 in simulation.

*The electrodes.* Detailed properties are mentioned in Table 2 and it has been used as a electrodes.

*The active layer.* The active layers of the simulated gas sensor is designed of two different thickness is 0.01 mm and 0.03 mm of Pd/rGO–SnO_2_ in the area of 5 × 1 mm on 1 × 1 gas sensor. To model such active layer in the COMSOL platform, it has predefined model and boundary condition, and the parameters and properties from the Tables 1 and 2 have been used.

The originally of the work and the simulation process hinge from the survey, also no one done any related work that simulated with/without active layers i.e., Pd/rGO–SnO_2_ based NO_x_ gas sensor. In general, a results from the COMSOL are used to simulate a chemical interaction and acting electronics structure and gas molecule dynamics in the solids and its full range of results can be seen in Supplementary Information. The simulation process using the software is time domain, also the output from the simulation after the absorption of gas molecules on the active layers can be easily readout in electrical parameters. In order to avoid any false alarming due to the environmental humidity factor, two identical films have been designed with/without active layers with various operating temperature starts from 25 °C to 95 °C and there will be differences in resultant of two films can be taken as a output. The changes in results of both with/without layers are due to the alter in surrounding impacts such as environmental temperature, humidity will be identical and not affect the output.

## Conclusions

In conclusion, a thin film SnO_2_ based Pd/rGO chemical gas sensor with IDE has been designed and simulated. The comparison effect of the chemical gas sensor with/without active layer has been studied first time using COMSOL Multiphysics. Significant detection is observed from the curve temperature Vs current under a low range of temperature between 20–100 °C gives NO_x_ sensing starts at 35 °C and reached its two maximum peaks one at 5 A/m^2^ when its at 70 °C, and another 4.5 A/m^2^ at 90 °C. From the simulated result, it expressed that this Pd/rGO based chemical gas sensor gives high sensing sensitivity. Evolved from outstanding sensing and basic merits of minimum power consumption, chemisorptions gas sensor combined ie., from the potential Vs current density norm, the active layer Of chemical gas sensor SnO_2_–Pd/rGO, the saturation state occurs at 5 V of 4.8A/m^2^ & SnO_2_–rGO sensor saturation states occur at 3 V of 4.5A/m^2^. Thin-film SnO_2_ deposited active layers of Pd/rGO with Au IDE. It provides an attractive alternative for high-performance NO_x_ sensing performance.

The selectivity of NO_x_ is improved after adding the active materials of rGO and Palladium on the layer of the active area in gas sensor. Also, the active material is chosen based on the literature survey study and its chemical and physical properties. By using these simulated results we can observe the sensitivity of the NO_x_ chemical gas sensor is directly proportional to applied load and temperature. This work can extend into practical study in future. In this work, the geometrical dimension & parameters of all these four sensors were analyzed and to obtain optimal performing active material gas sensors were preferred for practical application. Chemical gas sensor SnO_2_–Pd/rGO yield the higher sensible enthalpy 16,000 J/Kg at 95 °C. Temperature, surface charge density, potential difference, and current density norms. However optimum active materials also depend upon fabrication constraint and capacity. Among the four designs, tin dioxide with double active layers i.e., SnO_2_–Pd/rGO shows the highest sensitivity compared to other active material. Micro thin-film SnO_2_–Pd/rGO, SnO_2_–Pd, SnO_2_–rGO and SnO_2_ chemical gas sensors have been simulated and characterized. Based on the excellent sensible enthalpy performance for applied different operating temperatures. The SnO_2_, chemical gas sensor combined with Pd/rGO active material provides an attractive for high-performance NO_x_ sensing applications.

## Supplementary information

Supplementary Figures.

## Data Availability

The designing steps that support the findings of this study are openly available in at [https://www.comsol.com/]. Due to the nature of this designing and simulation research, authors of this study did not agree for their derived data to be shared publicly.
