# Supplementary material for: A computational study of a chemical gas sensor utilizing Pd–rGO composite on SnO2 thin film for the detection of NOx
Source: Sci Rep. 2021 Jan 13;11:970. doi: 10.1038/s41598-020-78586-7 (PMC7806668; doi:10.1038/s41598-020-78586-7)
Supplement: Supplementary file 1 — Supplementary Figures. [file 41598_2020_78586_MOESM1_ESM.pdf]

Supporting Information

A computational study of NO_x chemical gas sensor using Pd- rGO composite film

Akshya S[1,*] and Vimala Juliet A[2]

[*1] Research Scholar, Department of Electronics & Instrumentation Engineering, SRM Institute of Science & Technology, TN, India.

[2] Professor & Head, Department of Electronics & Instrumentation Engineering, SRM Institute of Science & Technology, TN, India

[*]Corresponding Author E-mail akshyas@srmist.edu.in

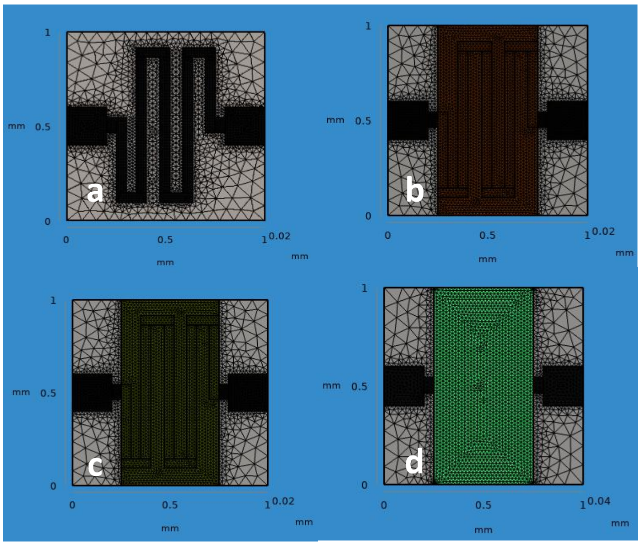

Figure S1. Normal meshing of the chemical gas sensor (a) SnO_2 (b) SnO_2-Pd (c) SnO_2-rGO and (d) SnO_2-Pd/rGO

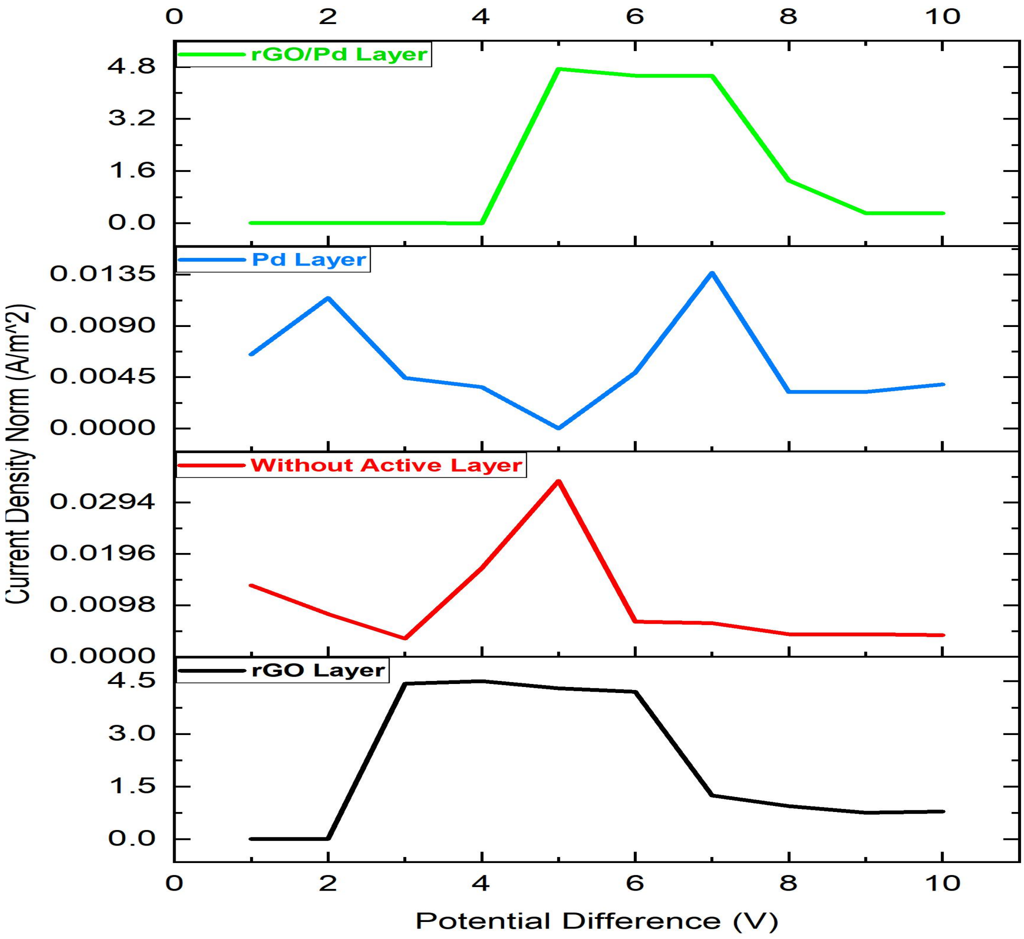

Figure S2. V-I response of Chemical gas sensor with and without active layers i.e, SnO_2 thin film - SnO_2-Pd - SnO_2-rGO - SnO_2-Pd/rGO

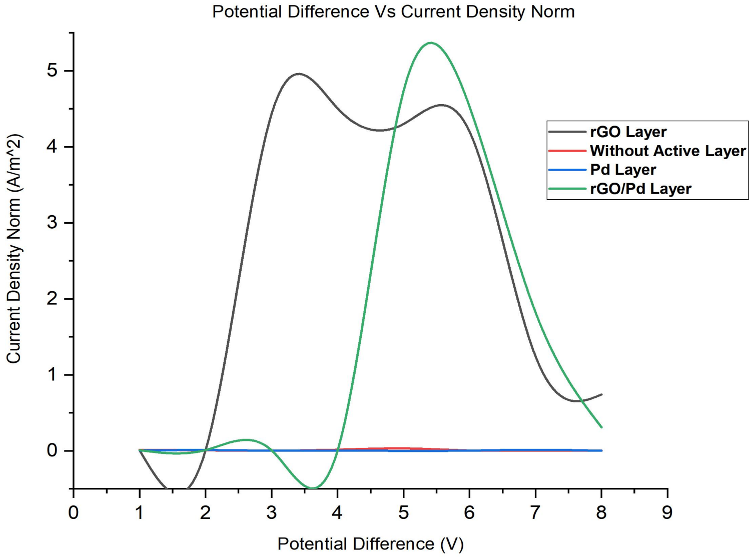

Figure S3. Effect and comparison of V-I response of all four chemical gas sensor i.e, SnO_2 thin film - SnO_2-Pd - SnO_2-rGO - SnO_2-Pd/rGO

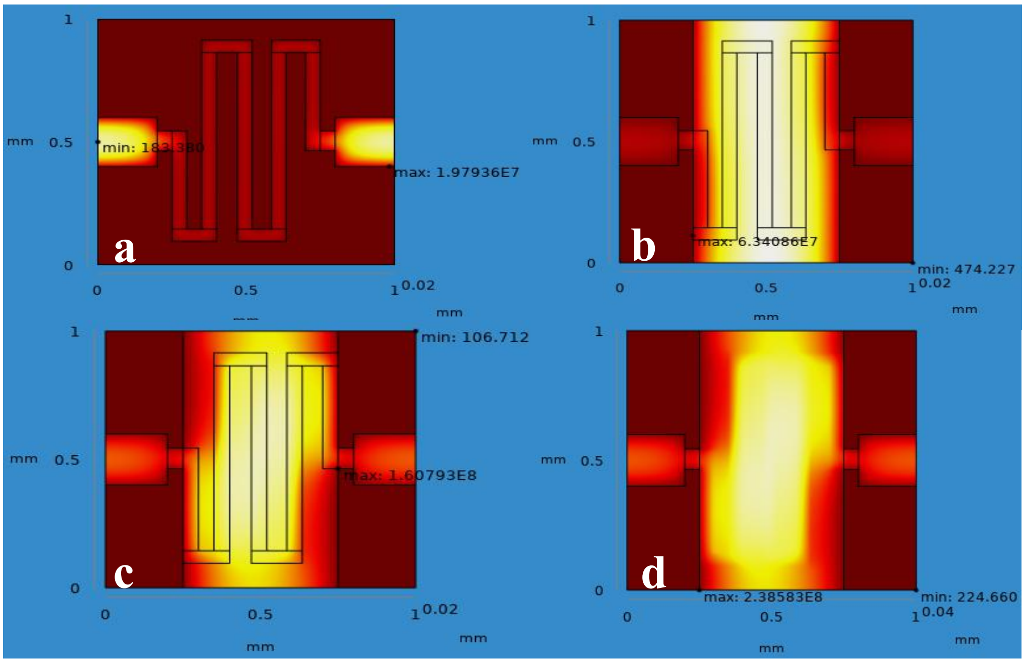

Figure S4. Temperature distribution on sensing layer of the chemical gas sensor : a)SnO_2 b) SnO_2-Pd c) SnO_2-rGO and d) SnO_2-Pd/rGO

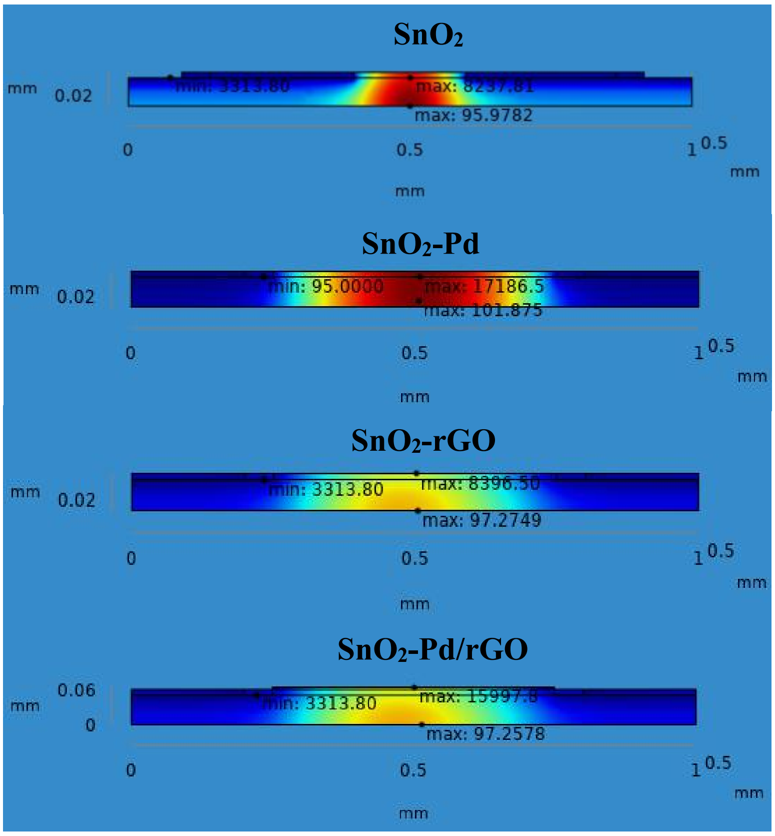

Figure S5. 3D Simulation NO_x sensing enthalpy flow over thin film chemical gas sensor: a) SnO_2 b)SnO_2-Pd c)SnO_2-rGO & d)SnO_2-Pd/rGO

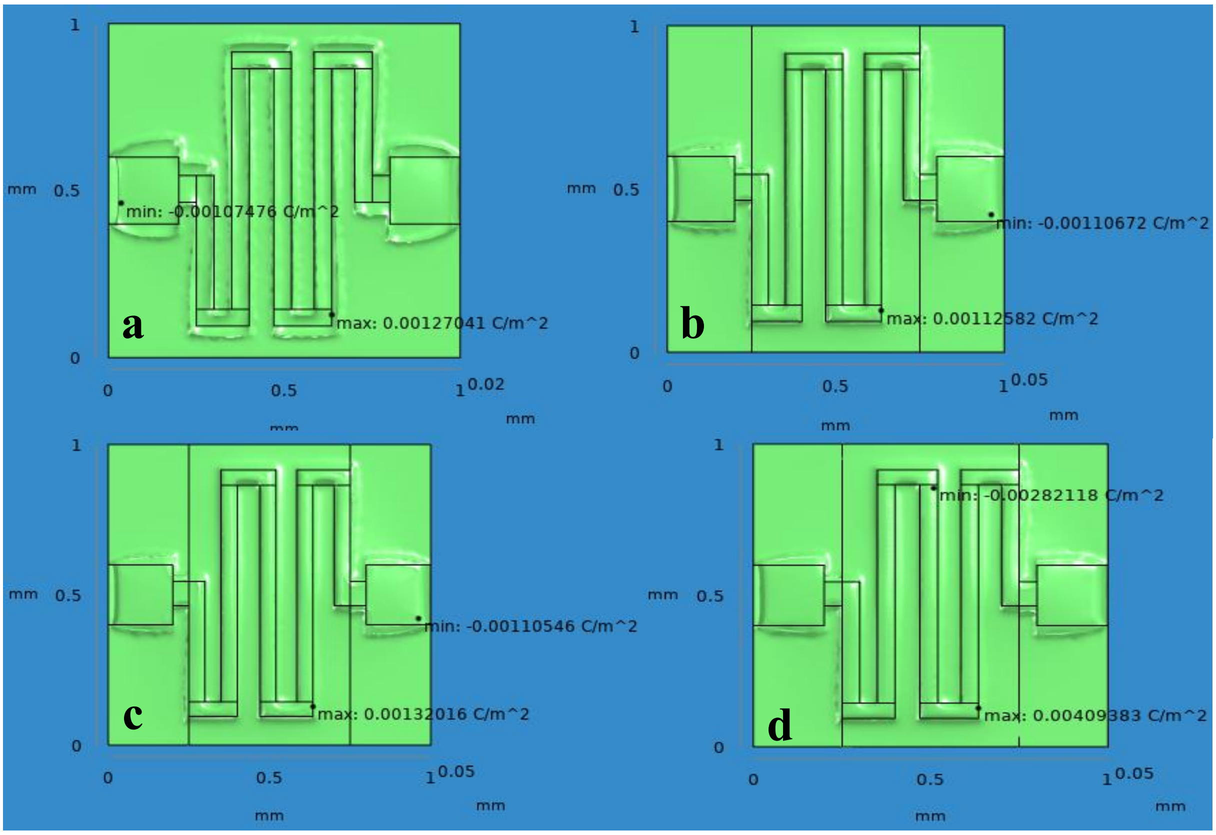

Figure S6. Stress distribution on the chemical gas sensor: a) SnO_2 b)SnO_2-Pd c)SnO_2-rGO and d)SnO_2-Pd/rGO

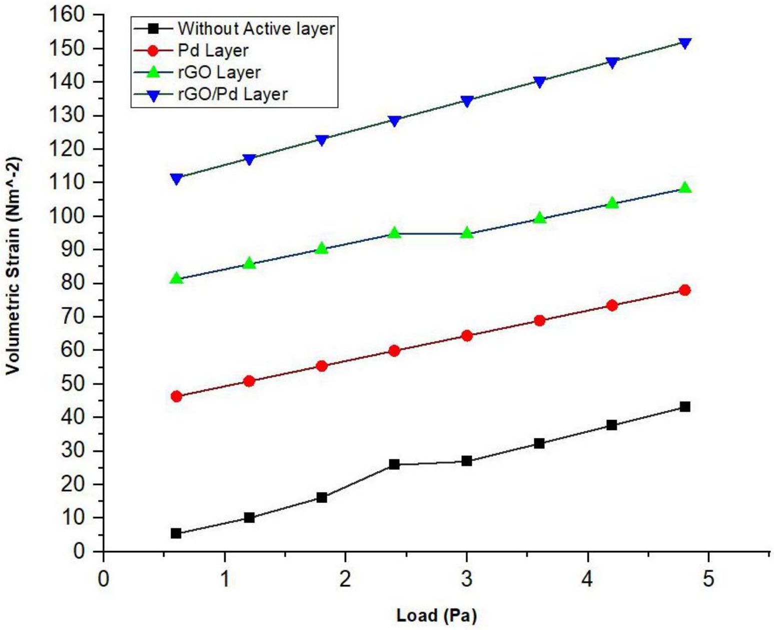

Figure S7. A Volumetric strain comparsion of all four design for the same applied load

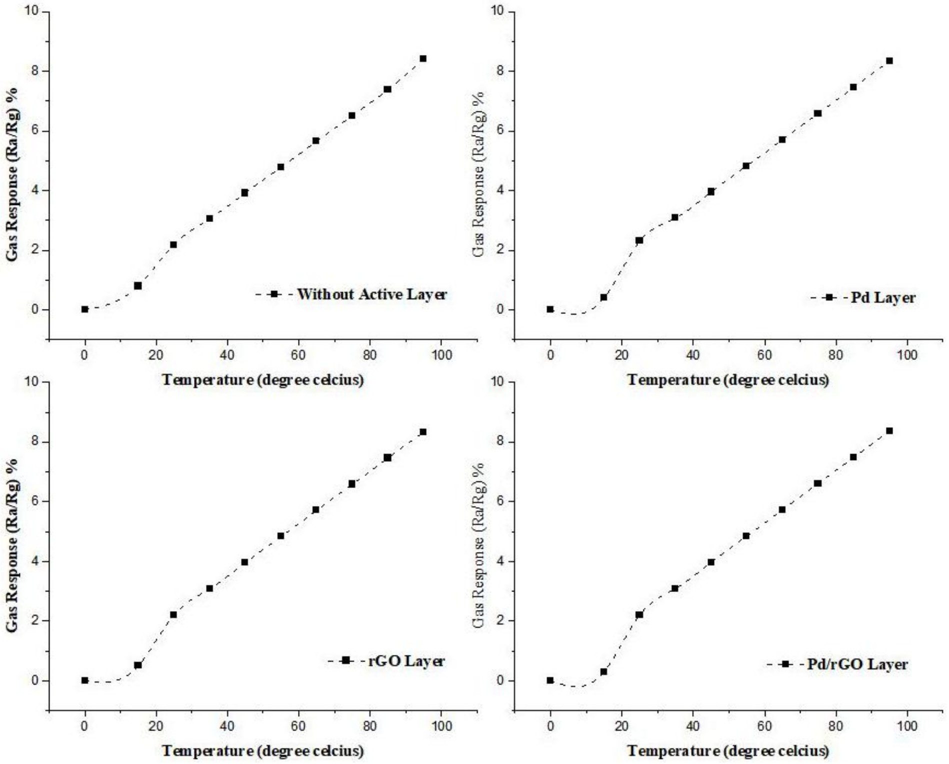

Figure S8. The simulated results of response at various working temperature.
